# A taxonomic monograph of the liphistiid spider genus *Heptathela*, endemic to Japanese islands

**DOI:** 10.3897/zookeys.888.34494

**Published:** 2019-11-11

**Authors:** Xin Xu, Hirotsugu Ono, Matjaž Kuntner, Fengxiang Liu, Daiqin Li

**Affiliations:** 1 College of Life Sciences, Hunan Normal University, 36 Lushan Road, Changsha 410081, Hunan Province, China Hubei University Wuhan China; 2 State Key Laboratory of Biocatalysis and Enzyme Engineering, Centre for Behavioural Ecology and Evolution, School of Life Sciences, Hubei University, 368 Youyi Road, Wuhan 430062, Hubei Province, China Hunan Normal University Changsha China; 3 Department of Zoology, National Museum of Nature and Science, 4-1-1 Amakubo, Tsukuba-shi, Ibaraki-ken, 305-0005, Japan National Museum of Nature and Science Tsukuba Japan; 4 Evolutionary Zoology Laboratory, Department of Organisms and Ecosystems Research, National Institute of Biology, Ljubljana, Slovenia National Institute of Biology Ljubljana Slovenia; 5 Evolutionary Zoology Laboratory, Biological Institute ZRC SAZU, Ljubljana, Slovenia Evolutionary Zoology Laboratory, Biological Institute ZRC SAZU Ljubljana Slovenia; 6 Department of Entomology, National Museum of Natural History, Smithsonian Institution, Washington, DC, USA National Museum of Natural History, Smithsonian Institution Washington United States of America; 7 Department of Biological Sciences, National University of Singapore, 14 Science Drive 4, 117543, Singapore National University of Singapore Singapore Singapore

**Keywords:** Heptathelinae, island endemism, Kyushu, Ryukyu archipelago, species delimitation, trapdoor spiders

## Abstract

Among the eight extant genera of primitively segmented spiders, family Liphistiidae, two are confined to East Asian islands, *Heptathela* Kishida, 1923 and *Ryuthela* Haupt, 1983. In this paper, a taxonomic revision of the genus *Heptathela* (Heptathelinae) from Kyushu and Ryukyu archipelago, Japan is provided. This study follows a multi-tier species delimitation strategy within an integrative taxonomic framework that is presented in a parallel paper, in which diagnosable lineages are considered as valid species. There, the initial hypothesis of species diversity (19) based on classical morphological diagnoses is tested with multiple species delimitation methods aimed at resolving conflict in data. This revision follows those analyses that converge on the species diversity of 20, which includes a pair of cryptic species that would have been undetected with morphology alone. After this revision, eight previously described species remain valid, two junior synonyms are proposed, and 12 new *Heptathela* species are described based on diagnostic evidence. To ease identification and to hint at putative evolutionary units, *Heptathela* is divided into three groups. The Kyushu group contains *H. higoensis* Haupt, 1983, *H. kikuyai* Ono, 1998, *H. kimurai* (Kishida, 1920), and *H. yakushimaensis* Ono, 1998; the Amami group contains *H. amamiensis* Haupt, 1983, *H. kanenoi* Ono, 1996, *H. kojima***sp. nov.**, *H. sumiyo***sp. nov.**, and *H. uken***sp. nov.**; and the Okinawa group contains *H. yanbaruensis* Haupt, 1983, *H. aha***sp. nov.**, *H. gayozan***sp. nov.**, *H. kubayama***sp. nov.**, *H. mae***sp. nov.**, *H. otoha***sp. nov.**, *H. shuri***sp. nov.**, *H. tokashiki***sp. nov.**, *H. unten***sp. nov.**, and *H. crypta***sp. nov.***Heptathela helios* Tanikawa & Miyashita, 2014 is not assigned to a species group. A combination of diagnostic tools augments the morphological diagnoses that, in isolation, would be prone to error in morphologically challenging groups of organisms.

## Introduction

**Figure 1.**
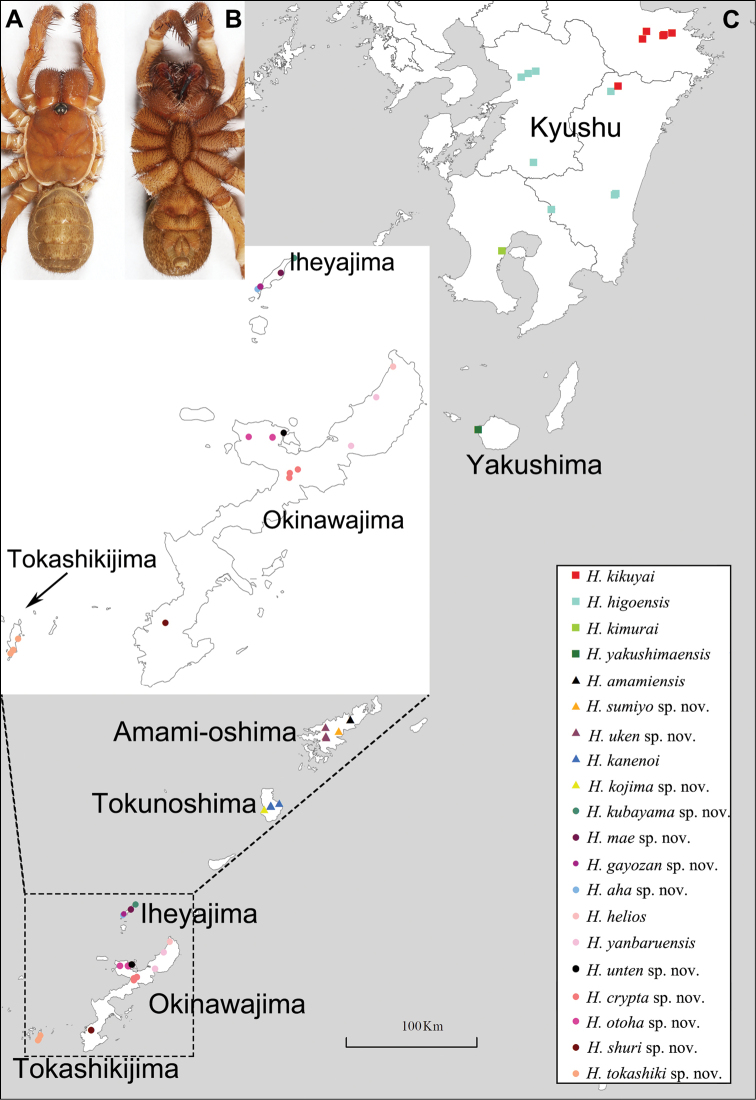
General somatic morphology of *Heptathela kimurai* (Kishida, 1920) **A** dorsal view **B** ventral view and map showing the sampling localities of *Heptathela* specimens from Kyushu to central Ryukyus (**C**).

The spider family Liphistiidae is the sole extant lineage within the ancient suborder Mesothelae known to possess a combination of plesiomorphic traits, for example, abdominal tergites (Fig. 1A), as well as spinnerets that have a mid-venter position (Fig. 1B). Compared to its sister clade that comprises all other spider families, liphistiids are genus- and species-poor, containing eight genera restricted to Southeast and East Asia (Xu et al. 2015a, b; World Spider Catalog 2019). Only two of these genera are purely insular, both being restricted to Japanese islands, and we focus here on the taxonomy of the nominal genus *Heptathela* Kishida, 1923 (Heptathelinae) (Fig. 1A, B).

Taxonomic limits of *Heptathela* were recently redefined (Xu et al. 2015b) as its taxonomic history that we briefly summarise here had been inconclusive. Kishida (1923) named the genus *Heptathela* for *Liphistius kimurai* Kishida, 1920 because its female had seven spinnerets, unlike the expected eight as in other species of *Liphistius* Schiödte, 1849. Although originally placed in Liphistiidae, *Heptathela* was once considered a family rank taxon, Heptathelidae (Petrunkevitch, 1939), again stressing the spinneret number difference with Liphistiidae. Only later, it was clarified that the spinneret number is variable among *Heptathela* species and not suitable for high level taxonomic diagnosis, even if it is stable in *Liphistius* (Yoshikura 1955; Haupt 1983, 2003; Xu et al. 2015a, b). Nonetheless, the genus name remained to be used for many “non-*Liphistius*” liphistiid lineages until 1983 when the second genus, *Ryuthela* Haupt, 1983, was added to the family Heptathelidae (Haupt 1983). This major clade was subsequently stabilised as the liphistiid subfamily Heptathelinae (Ono 2000; Xu et al. 2015b), but with several additional genera. Both Ono (2000) and Haupt (2003) relimited *Heptathela* to apply only to the species inhabiting the Japanese island Kyushu as well as the northern Ryukyu archipelago. These authors agreed that the species from China and Vietnam belonged to distinct genera. They disagreed, however, in the details. While Ono (2000) placed the mainland heptathelines into three genera (*Abcathela*, *Vinathela*, and *Songthela*), Haupt (2003) rejected *Abcathela* and *Vinathela*, erected instead *Nanthela* and *Sinothela*, and proposed *Songthela* as a synonym of the latter. To complete the full circle of this complex taxonomic history, Schwendinger and Ono (2011) reverted to only two heptatheline genera. According to these authors, all Chinese, Vietnamese and some Japanese species (not *Ryuthela*) were within *Heptathela* sensu lato, and the names *Nanthela* and *Sinothela* became its synonyms. All the above hypotheses were based exclusively on morphology. Using combined molecular and morphological data, we recently showed *Heptathela* sensu lato to be paraphyletic, and redefined the genus to comprise only the species from Kyushu and from the Ryukyus (Xu et al. 2015a, b).

*Heptathela* therefore does not occur in continental Asia (Xu et al. 2015b); instead, its species are endemic to Kyushu (six known species) as well as the northern and central islands of the Ryukyu archipelago (Tanikawa and Miyashita 2014; World Spider Catalog 2019), two are known from Amamioshima and Tokunoshima, and another two from Okinawajima. As emphasised in the above overview, most known *Heptathela* species have been delimited solely morphologically. The study by Tanikawa and Miyashita (2014) is an exception as they discovered one species, *H. helios* Tanikawa & Miyashita, 2014, and delimited *H. yanbaruensis* Haupt, 1983 and *H. helios* from Okinawa using genetic distances. Our recent molecular data used to test biogeographical hypotheses within East Asian margins, on the other hand, suggested that over ten *Heptathela* species exist on these islands (Xu et al. 2016).

We follow this evidence with a thorough taxonomic revision of *Heptathela*. As is the case in other liphistiids, *Heptathela* females usually lack clear morphologically diagnostic characters and furthermore exhibit considerable intraspecific variation. While males are more readily diagnosable morphologically, they are very rarely collected (Haupt 2003; Schwendinger and Ono 2011; Tanikawa 2013; Tanikawa and Miyashita 2014; Xu et al. 2015c, 2017). A purely morphological revision would thus be preliminary, or even erroneous, and be an underestimation of true species diversity (Xu et al. 2017). Therefore, an integrative taxonomic revision of this genus is a preferred alternative. We first establish species hypotheses for all available *Heptathela* specimens using morphological diagnoses. We then use the evidence from a range of molecular species delimitation analyses from a parallel study (Xu et al. 2019) to test and further diagnose these species, and discuss the benefits of such taxonomic approaches.

## Materials and methods

### Specimen acquisition

**Figure 2.**
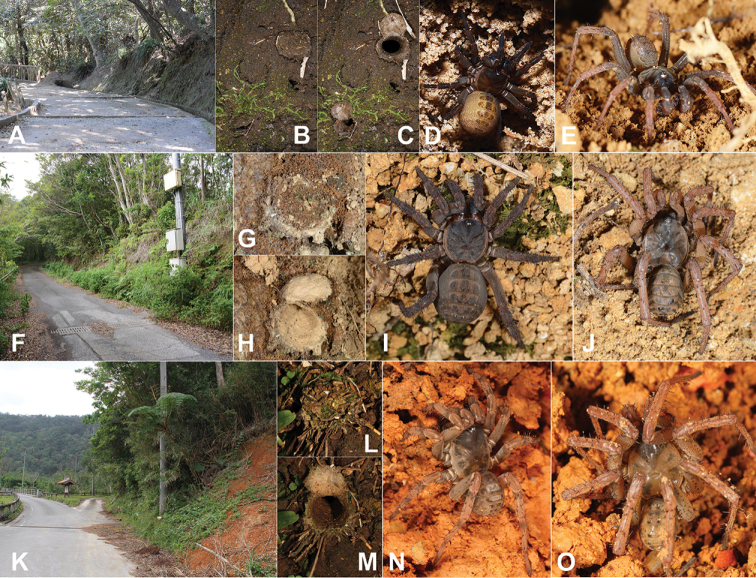
Microhabitats, trapdoors, and general somatic morphology of *Heptathela* Kishida, 1923 **A–E** Kyushu group: **A** microhabitat of *H. kimurai* (Kishida, 1920) at Shiroyama Park, Kyushu **B, C** open and closed trapdoor of *H. kimurai***D** female *H. kimurai***E** male *H. higoensis* (Haupt, 1983) **F–J** Amami group: **F** microhabitat of *H. sumiyo* sp. nov. at Sumiyo-cho, Amamioshima **G, H** open and closed trapdoor of *H. amamiensis* Haupt, 1983 **I** female *H. sumiyo* sp. nov. **J** male *H. sumiyo* sp. nov. **K–O** Okinawa group: **K** microhabitat of *H. yanbaruensis* Haupt, 1983 at Yona, Okinawajima **L, M** open and closed trapdoor of *H. yanbaruensis***N** female *H. yanbaruensis***O** male *H. yanbaruensis*.

Our original sampling, performed in the entire *Heptathela* range from Kyushu to the central Ryukyu archipelago, relied on the type locality information of each known *Heptathela* species, but also involved previously unexplored areas, focusing particularly on roadside habitats (Figs 1C, 2; for details of all specimens used in this study see Suppl. material 1: Table S1). We collected all specimens alive for initial check of their maturity status. We then fixed mature spiders in ethanol but retained all subadults alive to be reared to maturation. All mature specimens were subsequently preserved in 80% ethanol after being removed the right set of legs for the cryo-collection.

### Morphological examination

Specimens were morphologically examined under an Olympus SZX16 stereomicroscope and an Olympus BX51 compound microscope. Their genitalia were cleared in boiling 10% KOH for a few minutes to dissolve soft tissues. Unless noted otherwise, left palps were imaged. All measurements are reported in millimetres. Leg and palp measurements are given in the following order: total length (femur + patella + tibia + metatarsus + tarsus). The value ranges for cheliceral groove denticles, spinnerets and measurements in taxonomic descriptions are based on all the examined specimens.

Taxonomic descriptions using morphological characteristics follow our established methodology (Xu et al. 2015c, 2017). We use the following abbreviations throughout:

**ALE** anterior lateral eyes,

**AME** anterior median eyes,

**BL** body length,

**CL** carapace length,

**Co** conductor,

**CT** contrategulum,

**CW** carapace width,

**D** depression,

**E** embolus,

**OL** opisthosoma length,

**OW** opisthosoma width,

**PC** paracymbium,

**PLE** posterior lateral eyes,

**PME** posterior median eyes,

**RC** receptacular cluster,

**T** tegulum.

Museum abbreviations:

**CBEE** Centre for Behavioural Ecology and Evolution, School of Life Sciences, Hubei University, Wuhan, China;

**NMNS**National Museum of Nature and Science, Japan;

**MCZ**National Zoological Museum of China, Institute of Zoology, Chinese Academy of Sciences, Beijing, China;

**ZMH**Museum of Comparative Zoology, Harvard University, Cambridge, USA;

**ZMH** Zoological Museum Hamburg, Germany.

Voucher specimens are deposited at CBEE, and the type specimens will be deposited in NMNS and MCZ.

### Integrative taxonomic framework

The integrative taxonomic framework considers diagnosable lineages as potentially valid species. Our taxonomic approach is based on morphological diagnoses that provide an initial species hypothesis (19 species). This is then further tested within a parallel, molecular species delimitation study (Xu et al. 2019). The parallel study amplified two molecular loci, the classical animal barcoding region (COI) as well as the nuclear ITS2 for most sampled specimens, and analysed those data using a multi-tier species delimitation strategy. In tier 1 analysis, six different sequence-based species delimitation methods using the full data matrix containing 180 samples are used to test the here proposed number of morphologically delimited species of *Heptathela*. The fully congruent species supported by all the methods are included within the final species counts, but only the conflicting species enter the tier 2 analysis in which more genetic markers are added for the samples that represent those conflicting lineages and multi-locus coalescent-based delimitation methods are used to test competing species models derived from tier 1 analysis. If conflict persists, our integrative approach re-evaluates DNA barcode gaps to delimit the competing species models derived from tier 2 analysis. The additional molecular diagnostic evidence, species specific nucleotide substitution information in the animal barcoding gene region (i.e. the standard alignment of COI) (Brower 2010; Cook et al. 2010; Planas and Ribera 2015), augments the morphological species diagnoses that in isolation would be prone to error.

The aligned DNA barcode gene COI is deposited in the Dryad Digital Repository.

## Results and discussion

Of the total species diversity of 20, eight previously described species remain valid, two junior synonyms are proposed, and 12 new *Heptathela* species are discovered and described. This taxonomic revision thus represents a 137.5% increase in species diversity of the genus, drawing parallels with prior species richness underestimation found also in other liphistiid genera, e.g., *Ganthela* (Xu et al. 2015c) and *Ryuthela* (Xu et al. 2017).

**Figure 3.**
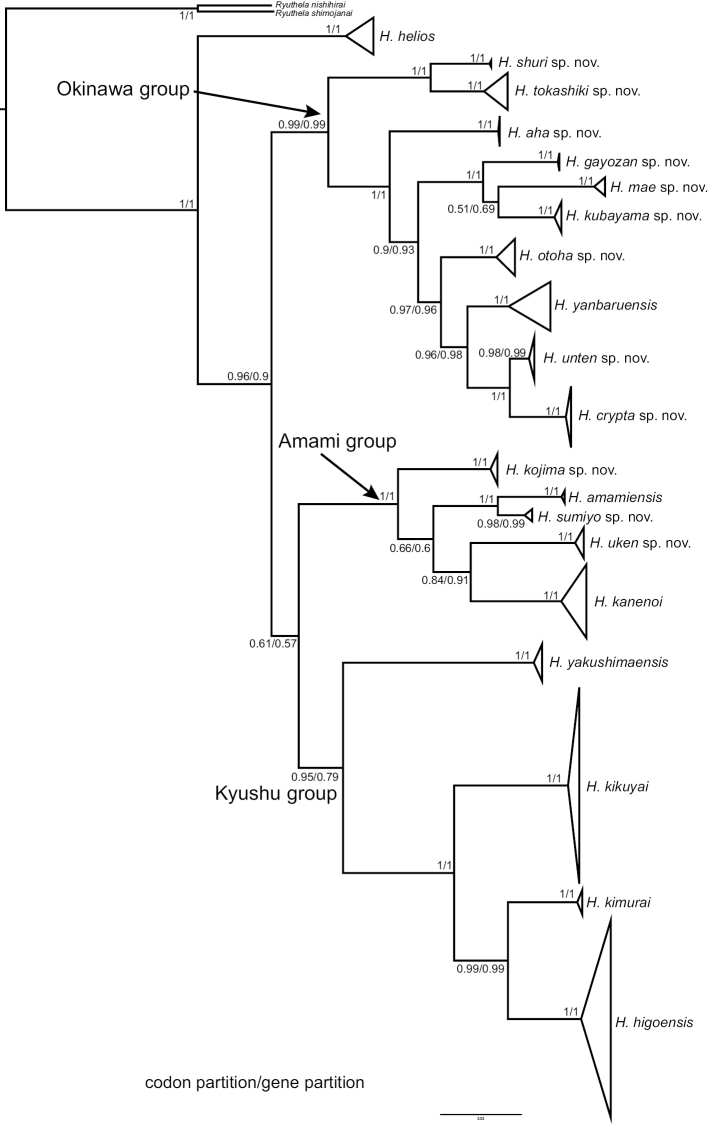
A simplified species-level phylogeny derived from the BI tree in the parallel paper (Xu et al. 2019) showing three species groups.

To ease identification and to hint at putative evolutionary units, we divide *Heptathela* into the Kyushu, the Amami, and the Okinawa groups, each of which contains four, five, and ten species, respectively (Fig. 3). This division of groups is mostly based on species’ morphology and geographical distributions, but also heeds the phylogeny. Hence, to avoid paraphyly of Okinawa group, we do not assign *H. helios* from northern Okinawajima to a group as it is sister to all the other *Heptathela* species (Fig. 3).

### Taxonomy of Heptathela

#### Genus *Heptathela* Kishida, 1923

**Type species.**

**Figure 4.**
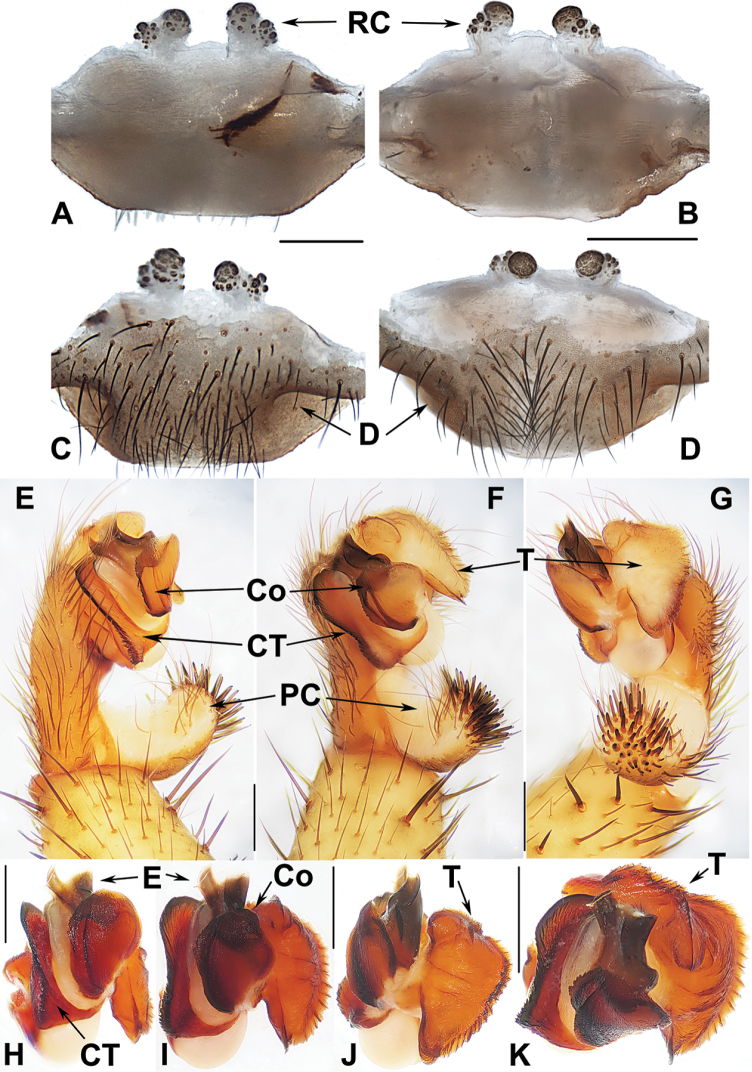
Male and female genital anatomy of *Heptathela higoensis* Haupt, 1983 **A, C** 3472 (short for XUX-2013-472) **B, D** 3435 **E–G** 3365 **H–K** 3381 **A, B** vulva dorsal view **C, D** vulva ventral view **E** palp prolateral view **F** palp ventral view **G** palp retrolateral view **H–K** palp distal view; 3365: Hitoyoshi Ruins Park, Kumamoto; 3472: Takachihokawara, Kagoshima; 3435: Mukoyama, Miyazaki; 3381: Kozomo, Kumamoto. Scale bar: 0.5 mm.

**Diagnosis.***Heptathela* males differ from *Liphistius* males by lacking the tibial apophysis, and from males of all other Heptathelinae genera by possessing a leaf-shaped conductor (Fig. 4E, F, H), a wide and thumb-shaped embolus (Fig. 4F, I, K), and a wide tegulum with rugose margin (Fig. 4J, K). *Heptathela* females differ from *Liphistius* females by paired receptacular clusters, and from females of all other Heptathelinae genera by paired depressions on the ventro-lateral part of the genital atrium (Fig. 4C, D), and by the paired receptacular clusters with the main and secondary, lateral, irregular receptacular clusters (Fig. 4A–D). 

**General description.** All the 20 species share the following characteristics: sternum narrow, longer than wide; a few short pointed hairs running over ocular mound in a longitudinal row; chelicerae robust with promargin of cheliceral groove with variable sized denticles (the number of denticles is presented under each species description); legs with strong hairs and spines, and male legs distinctly longer than female legs; opisthosoma with 12 tergites, and the fifth tergite is the largest in all the species except in *H. amamiensis*, *H. sumiyo* sp. nov., and *H. uken* sp. nov., in which the fourth tergite is the largest. 

##### The Kyushu group

**Diagnosis.** The males of the Kyushu group differ from those of the other two groups by the nearly rectangular contrategulum whose two proximal thirds are serrated (Fig. 4H, I), whereas it is semi-elliptic in the others. The Kyushu group females resemble those of the Okinawa group, but differ from those of the Amami group by distinctly paired depressions on the ventro-lateral part of the genital atrium (Fig. 4C, D).

**Monophyly.** The Bayesian analyses based on concatenated two genes and two partitions (for details, see Xu et al. 2019) inferred the same topology, which supports the Kyushu group monophyly (posterior probability, hereafter PP = 0.95/0.79) (Fig. 3).

**Composition.***H. higoensis* Haupt, 1983, *H. kikuyai* Ono, 1998, *H. kimurai* (Kishida, 1920), and *H. yakushimaensis* Ono, 1998.

**Distribution.** Kyushu and Yakishima (Fig. 1C).

###### Heptathela higoensis

Taxon classificationAnimaliaAraneaeLiphistiidae

Haupt, 1983

342FD344-DA73-5200-BD2F-DF1A41B67EE7

Figs 4 , 5

Heptathela kimurai higoensis Haupt, 1983: 283 (holotype: male, from Kumamoto, Kyushu, Japan, collected by M. Yoshikura on 27 September 1973, deposited in ZMH, examined); Haupt 2003: 69. Heptathela higoensis: Ono 1998: 16; Ono 2009: 80; Ono and Ogata 2018: 26, 479.
Heptathela nishikawai Ono, 1998: 19 (holotype: female, from Hitoyoshi-shi, Kumamoto-ken, Kyushu, Japan, collected by H. Ono on 19 November 1996, deposited in NMNS, examined); Ono 2009: 83; syn. nov.
Heptathela yaginumai Ono, 1998: 20 (holotype: female, from Honjo, Kunitomi-cho, Higashimorokata-gun, Miyazaki-ken, Kyushu, Japan, collected by T. Yaginuma on 18 June 1949, deposited in NMNS, examined); Ono 2009: 81. syn. nov.

####### Diagnosis.

Males of *H. higoensis* can be distinguished from those of *H. kikuyai* by one of the embolus peaks being longer than the other (Fig. 4H, I, K) and by the slightly blunt tegular marginal apophysis (Fig. 4J), and from those of *H. yakushimaensis* by the conductor with the weakly serrated prolateral margin (Fig. 4F, H, K). Females of *H. higoensis* can be distinguished from those of *H. kimurai* by the wide and flat dorso-posterior part of the genital area (Fig. 4A, B), and from those of *H. kikuyai* and *H. yakushimaensis* by the inner receptacular clusters that are larger than the outer ones (Fig. 4A–D). Moreover, *H. higoensis* differs from all other Kyushu group *Heptathela* species by the following unique nucleotide substitutions in the standard DNA barcode alignment: G (140), C (146), A (179), C (251), C (257), C (263), G (272), A (326), C (332), T (350), G (479), G (569), C (572), A (578), G (596), G (632), G (641).

####### Description.

**Males** (*N* = 11). Carapace yellow brown; opisthosoma light brown, with dark brown tergites close to each other; cheliceral groove with 11–13 denticles; 7 or 8 spinnerets. Measurements: BL 8.80–11.00, CL 4.40–5.25, CW 4.10–4.90, OL 4.40–5.60, OW 2.90–3.40; ALE > PLE > PME > AME; leg I 11.75 (3.45 + 1.50 + 2.60 + 2.70 + 1.50), leg II 12.40 (3.40 + 1.60 + 2.50 + 3.10 + 1.80), leg III 13.30 (3.20 + 1.60 + 2.40 + 3.80 + 2.30), leg IV 16.80 (4.40 + 1.20 + 3.30 + 5.20 + 2.70).

***Palp*.** Prolateral side of paracymbium unpigmented and unsclerotised, numerous setae and spines at the tip of paracymbium (Fig. 4E–G). Contrategulum with serrated margin proximally and smooth margin distally (Fig. 4I, K). Tegulum wide, the dorsal extension of terminal apophysis and marginal apophysis with a serrated margin retrolaterally (Fig. 4J, K). Conductor wide and with an apical tooth and a deep fold (Fig. 4H, K). Embolus with two peaks, one peak longer than the other, and with a curved margin retrolaterally (Fig. 4H–K).

**Females** (*N* = 43). Carapace and opisthosoma colour as in male; cheliceral groove with 11–16 pronounced denticles; tergites similar to male; 7–8 spinnerets. Measurements: BL 8.00–12.80, CL 4.30–6.10, CW 3.80–5.57, OL 4.10–6.50, OW 2.70–4.90; ALE > PLE > PME > AME; palp 8.20 (2.70 + 1.50 + 1.70 + 2.30), leg I 8.80 (2.80 + 1.75 + 1.70 + 1.55 + 1.00), leg II 8.93 (2.75 + 1.60 + 1.55 + 1.83 + 1.20), leg III 9.30 (2.70 + 1.60 + 1.50 + 2.20 + 1.30), leg IV 13.25 (3.65 + 1.80 + 2.40 + 3.40 + 2.00).

***Female genitalia*.** A pair of depressions on the ventro-lateral part of genital atrium (Fig. 4C, D). Paired receptacular clusters along the anterior margin of bursa copulatrix, divided into two parts, the inner main part forming a large granulate tubercle, with short genital stalks, the outers with several small granules (Fig. 4).

####### Remarks.

**Figure 5.**
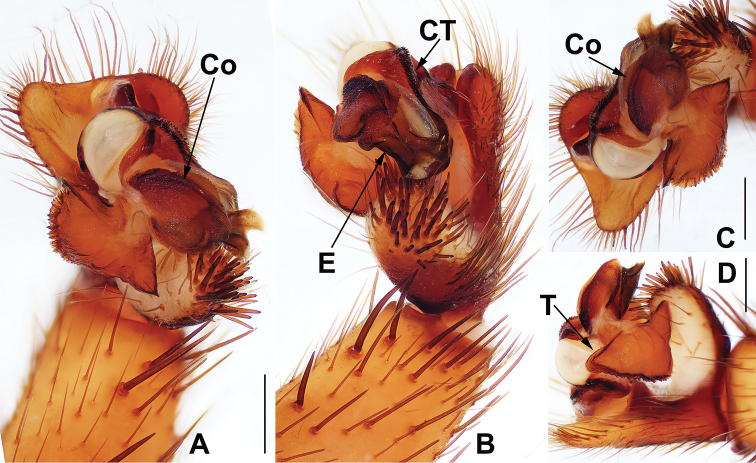
Male holotype genital anatomy of *Heptathela higoensis* Haupt, 1983 (ZMH 21751) **A** palp ventral view **B** palp retrolateral view **C, D** palp distal view. Scale bar: 0.5 mm.

We examined the male holotype of *H. higoensis* (Fig. 5) and identified the species as *H. higoensis* even though the bulb of holotype male is relatively distorted compared to the specimens we collected. After we examined 11 males and 43 females collected at the type localities of *H. higoensis*, *H. nishikawai* and *H. yaginumai*, and compared the holotypes of *H. higoensis*, *H. nishikawai* and *H. yaginumai* with our specimens, we proposed synonymy of *H. nishikawai* and *H. yaginumai* with *H. higoensis* based on their genital morphology, molecular species delimitation (Xu et al. 2019), and intraspecific genetic distances, 0–1.19% (K2P) and 0–1.18% (*p*-distance) among 43 specimens, although females exhibit a considerable intraspecific variation in genitalia.

####### Material examined.

JAPAN · 1 ♂, 8 ♀♀; Kyushu, Kumamoto-ken, Hitoyoshi-shi, Fumotomachi, Hitoyoshi Ruins Park; 32.21N, 130.77E; alt. 140 m; 18 September 2013; D. Li and B. Wu leg.; XUX-2013-365 (♂ matured 19 July 2014 at CBEE), XUX-2013-360 to 364, 366 to 368 · 2 ♂♂, 5 ♀♀; Kyushu, Kumamoto-ken, Kumamoto-shi, Tatsutayama, Tatsuta National Park; 32.82N, 130.73E; alt. 60 m; 19 September 2013; D. Li and B. Wu leg.; XUX-2013-370 to 379 · 4 ♂♂, 4 ♀♀; Kyushu, Kumamoto-ken, Kumamoto-shi, Higashi-ku, Kozono 1-chome; 32.84N, 130.78E; alt. 100 m; 19 September 2013; D. Li and B. Wu leg.; XUX-2013-380 to 389 (XUX-2013-381, ♂ matured 2 August 2014 at CBEE) · 1 ♂, 3 ♀♀; Kyushu, Kumamoto-ken, Kumamoto-shi, Kasuga, Hanaokayama; 32.80N, 130.68E; alt. 120 m, 19 September 2013; D. Li and B. Wu leg.; XUX-2013-390 to 393 · 3 ♀♀; Miyazaki-ken, Nishiusuki-gun, Takachiho-cho, Mukoyama; 32.70N, 131.30E; alt. 320 m; 22 September 2013; D. Li and B. Wu leg.; XUX-2013-435 to 441 · 1 ♂, 3 ♀♀; Miyazaki-ken, Higashimorokata-gun, Kunitomi-cho, Honjo 11960-1; 32.00N, 131.34E; alt. 30 m; 23 September 2013; D. Li and B. Wu leg.; XUX-2013-456 (♂, matured 19 July 2014 at CBEE), XUX-2013-449 to 451 · 1 ♂, 12 ♀♀; Miyazaki-ken, Higashimorokata-gun, Kunitomi-cho, Honjo 4191; 31.98N, 131.33E; alt. 30 m; 23 September 2013; D. Li and B. Wu leg.; XUX-2013-457 to 467C · 1 ♀; Miyazaki-ken, Nishimorokata-gun, Takaharu-cho, Kamamuto, Lake Miike; 31.89N, 130.96E; alt. 360 m; 23 September 2013; D. Li and B. Wu leg.; XUX-2013-468 · 1 ♂, 4 ♀♀; 2 Kagoshima-ken, Kirishima-shi, Kirishima, Takachihokawara; 31.89N, 130.89E; alt. 960 m; 23 September 2013; D. Li and B. Wu leg.; XUX-2013-471 (♂, matured 8 June 2014 at CBEE), XUX-2013-472 to 474, 476.

####### Distribution.

The species is known from the following prefectures on the Japanese island Kyushu: Kumamoto-ken (Hitoyoshi-shi and Kumamoto-shi), Miyazaki-ken (Nishiusuki-gun, Higashimorokata-gun and Nishimorokata-gun), Kagoshima-ken (Kirishima-shi) (Fig. 1C).

###### Heptathela kikuyai

Taxon classificationAnimaliaAraneaeLiphistiidae

Ono, 1998

90060E34-E05C-5574-AA14-B6C27D7BF304

Fig. 6

Heptathela kikuyai Ono, 1998: 16 (holotype: male, from Mt. Gozaga-dake, 20 km south of Oita-shi, Oita-ken, Kyushu, Japan, collected by N. Kikuya on 13 September 1979, deposited in NMNS, examined); Ono 2009: 81; Schwendinger and Ono 2011: 613; Ono and Ogata 2018: 26, 479.

####### Diagnosis.

**Figure 6.**
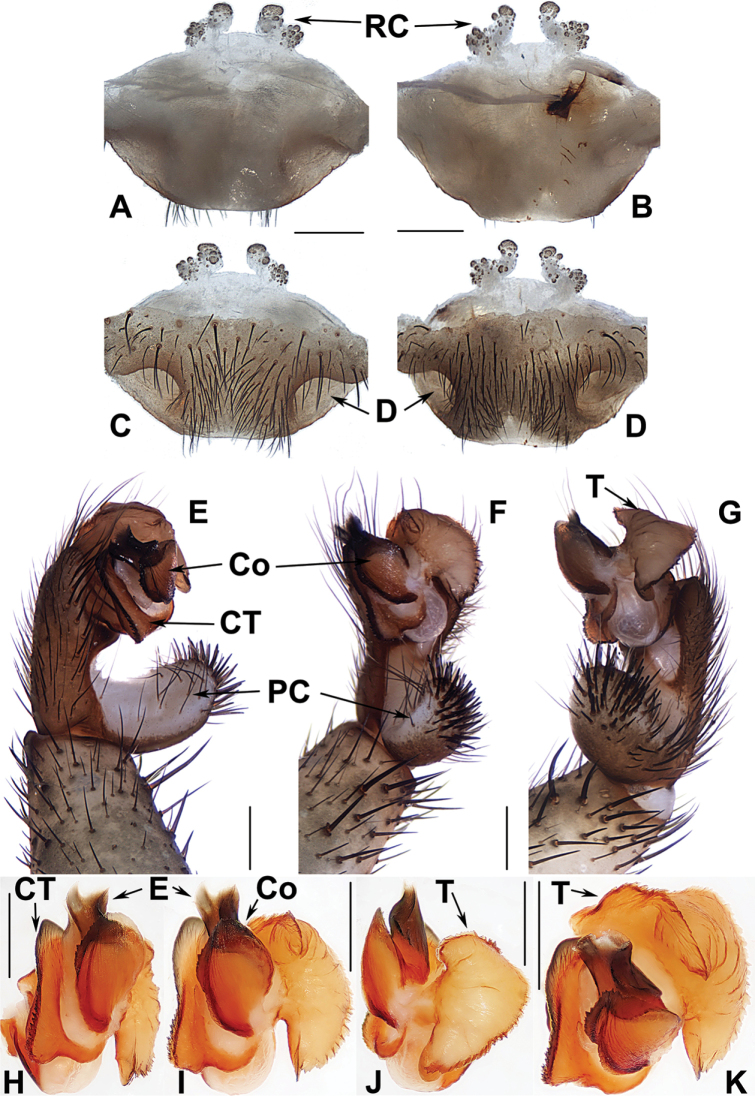
Male and female genital anatomy of *Heptathela kikuyai* Ono, 1998 **A, C** 3409 (short for XUX-2013-409) **B, D** 3401 **E–K** 3405 **A, B** vulva dorsal view **C, D** vulva ventral view **E** palp prolateral view **F** palp ventral view **G** palp retrolateral view **H–K** palp distal view; 3405, 3409: Mt. Gozaga-dake, Oita-ken; 3401: Onomachi, Oita-ken. Scale bar: 0.5 mm.

Males of *H. kikuyai* differ from those of *H. higoensis* by the two embolus peaks of a similar height, and the hooked tegular marginal apophysis (Fig. 6J, K), and from those of *H. yakushimaensis* by only a weakly serrated prolateral margin of the conductor (Fig. 6H, I). Females of *H. kikuyai* differ from those of all other Kyushu group *Heptathela* species by the inner receptacular clusters with longer and slender stalks (Fig. 6A–D). Moreover, *H. kikuyai* differs from all other Kyushu group *Heptathela* species by the following unique nucleotide substitutions in the standard DNA barcode alignment: G (11), A (33), C (41), C (59), C (68), A (83), A (89), C (116), T (194), A (212), T (222), A (239), A (251), C (260), T (276), T (287), C (305), C (329), A (345), T (365), C (470), G (491), T (546), T (578), T (581), G (635), C (656).

####### Description.

**Males** (*N* = 5). Carapace and opisthosoma brown, with dark-spotted tergites; cheliceral groove with 10–12 denticles; 6 or 7 spinnerets. Measurements: BL 7.90–9.40, CL 4.00–5.05, CW 3.55–4.30, OL 3.90–4.60, OW 2.60–3.20; ALE > PLE > PME > AME; leg I 13.65 (3.90 + 1.90 + 2.75 + 3.30 + 1.80), leg II 13.35 (3.45 + 1.60 + 2.65 + 3.55 + 2.10), leg III 14.40 (3.30 + 1.50 + 2.75 + 4.45 + 2.40), leg IV 19.25 (4.85 + 1.90 + 3.35 + 6.00 + 3.15).

***Palp*.** Prolateral side of paracymbium unpigmented and unsclerotised, numerous setae and spines at the tip of paracymbium (Fig. 6E–G). Contrategulum with serrated margin proximally and smooth margin distally (Fig. 6H, I, K). Tegulum wide, the dorsal extension of terminal apophysis and hooked marginal apophysis of tegulum with a serrated margin retrolaterally (Fig. 6J, K). Conductor wide and with an apical tooth and a deep fold (Fig. 6I, K). Embolus with two peaks at the slightly same level and a curved margin retrolaterally (Fig. 6H–K).

**Females** (*N* = 30). Carapace and opisthosoma as in male; cheliceral groove with 12–17 pronounced denticles; tergites similar to male; 6–8 spinnerets. Measurements: BL 8.55–15.10, CL 4.30–7.90, CW 3.80–6.70, OL 4.40–8.75, OW 2.80–6.10; ALE > PLE > PME > AME; palp 9.15 (3.25 + 1.60 + 1.90 + 2.40), leg I 10.30 (3.30 + 1.90 + 1.90 + 2.00 + 1.20), leg II 10.15 (3.10 + 1.80 + 1.85 + 2.10 + 1.30), leg III 10.80 (3.00 + 1.80 + 1.80 + 2.60 + 1.60), leg IV 15.80 (4.50 + 2.20 + 2.80 + 4.10 + 2.20).

***Female genitalia*.** A pair of depressions on the ventro-lateral part of genital atrium. Paired receptacular clusters along the anterior margin of bursa copulatrix, indistinctly divided into two parts, with many small granules, inner ones with longer and slender genital stalks (Fig. 6A–D).

####### Material examined.

JAPAN · 6 ♀♀; Oita-ken, Bungoono-shi, Totoki, Onomachi; 33.06N, 131.52E; alt. 240 m; 20 September 2013; D. Li and B. Wu leg.; XUX-2013-394 to 396, 399 to 401 · 2 ♂♂, 4 ♀♀; Oita-ken, Bungoono-shi, Onomachi-Ando, Mt. Gozaga-dake; 33.11N, 131.55E; alt. 630 m; 20 September 2013; D. Li and B. Wu leg.; XUX-2013-404, 405, 408, 409, 411, 412 (405, ♂ matured 2 May 2014 at CBEE) · 2 ♂♂, 6 ♀♀; Oita-ken, Usuki-shi, Takeyama; 33.10N, 131.72E; alt. 80 m; 20 September 2013; D. Li and B. Wu leg.; XUX-2013-414, 416 to 422 · 1 ♂, 8 ♀♀; Oita-ken, Usuki-shi, Inukai-machi Sasamuta; 33.09N, 131.67E; alt. 120 m; 21 September 2013; D. Li and B. Wu leg.; XUX-2013-424 to 430, 432, 433 · 6 ♀♀; Miyazaki-ken, Nishiusuki-gun, Takachiho-cho, Iwato; 32.73N, 131.35E; alt. 400 m; 22 September 2013; D. Li and B. Wu leg.; XUX-2013-442 to 444, 446 to 448.

####### Distribution.

The species is known from the following prefectures on the Japanese island Kyushu: Oita-ken (Bungoono-shi and Usuki-shi) and Miyazaki-ken (Nishiusuki-gun) (Fig. 1C).

###### Heptathela kimurai

Taxon classificationAnimaliaAraneaeLiphistiidae

(Kishida, 1920)

51999F5A-A329-5810-A798-EBC5826C670B

Fig. 7

Liphistius kimurai Kishida, 1920: 362 (holotype: female, from Shiroyama, Kagoshima, Kyushu, Japan, collected by A. Kimura in October 1920, lost in the Science College Museum of the Tokyo Imperial University (Haupt, 1983); neotype: male, from the same locality as for the original type specimen, collected by J. Haupt on 21 March 1982, matured in August 1982, deposited in ZMH, but the neotype may be lost according to Dunlop et al. 2014).
Heptathela kimurai Kishida, 1923: 236; Bristowe 1933: 1030; Sawaguti and Ozi 1937: 116 (partly); Yaginuma 1954: 15; Yaginuma 1955: 35; Yaginuma 1960: 19; Yaginuma 1971: 19; Gertsch and Platnick 1979: 5; Yaginuma 1979: 1; Yaginuma 1980: 44; Haupt 1983: 283; Yoshikura 1983: 63; Haupt 1984: 163; Yaginuma 1986: 1; Yoshikura 1987: 148; Chikuni 1989b: 18; Ono 1998: 14; Yoo and Kim 2002: 27; Haupt 2003: 69; Ono 2009: 81; Schwendinger and Ono 2011: 614; Ono and Ogata 2018: 25, 479.

####### Diagnosis.

**Figure 7.**
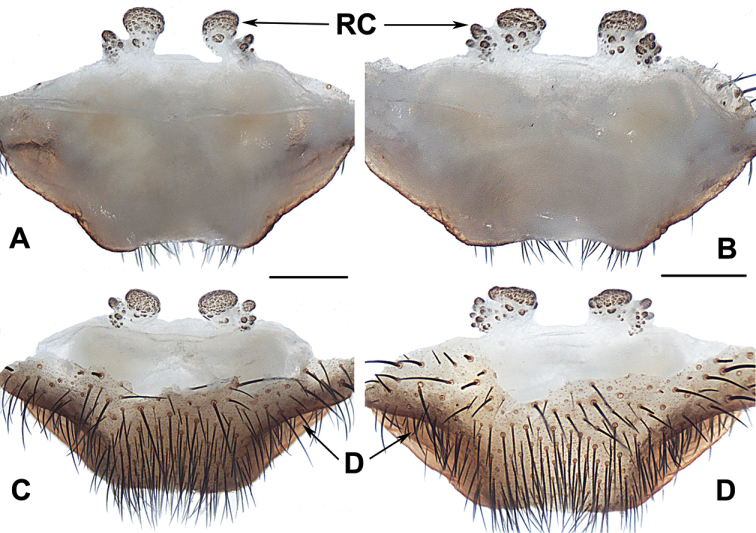
Female genital anatomy of *Heptathela kimurai* (Kishida, 1920) **A, C** 3351 (short for XUX-2013-351) **B, D** 3359 **A, B** vulva dorsal view **C, D** vulva ventral view. Scale bar: 0.5 mm.

Females of *H. kimurai* resemble *H. higoensis* females but differ by a slightly curved dorso-posterior margin of the genital area (Fig. 7A, B). Moreover, *H. kimurai* differs from all other Kyushu group *Heptathela* species by the following unique nucleotide substitutions in the standard DNA barcode alignment: C (26), T (98), G (191), G (194), G (206), C (215), T (251), T (278), G (293), C (362), T (366), G (443), T (449), C (452), G (506), G (521), C (527), G (548), T (572), C (599), A (615), C (638).

####### Description.

**Females** (*N* = 9). Carapace yellow brown; opisthosoma brown, with brown tergites close to each other; cheliceral groove with 12–14 vestigial denticles; seven spinnerets. Measurements: BL 9.30–13.80, CL 4.88–6.30, CW 4.10–5.50, OL 4.80–7.30, OW 3.45–5.40; ALE > PLE > PME > AME; palp 7.77 (2.57 + 1.50+ 1.60 + 2.10), leg I 9.15 (2.85 + 1.70 + 1.75 + 1.65 + 1.20), leg II 8.88 (2.65 + 1.65 + 1.55 + 1.88 + 1.15), leg III 9.63 (2.70 + 1.70 + 1.60 + 2.30 + 1.33), leg IV 14.08 (3.88 + 1.90 + 2.50 + 3.60 + 2.20).

***Female genitalia*.** A pair of depressions on the ventro-lateral part of genital atrium. Paired of receptacular clusters along the anterior margin of bursa copulatrix, divided into two parts, the inner main part forming a large granulate tubercle, the outer part with several small granules (Fig. 7A–D).

**Male**: unknown.

####### Remarks.

We could not examine the presumably lost neotype male (Dunlop et al. 2014). The male is therefore unknown. The non-topotypical male described as *H. kimurai* by Ono (1998, 2009) is excluded here as we are not sure that the specimen was collected from the type locality although it was obtained in Kagoshima-shi.

####### Material examined.

JAPAN · 9 ♀♀; Kagoshima-ken, Kagoshima-shi, Shiroyama-cho, Shiroyama Park; 31.60N, 130.55E; alt. 100 m; 18 September 2013; D. Li and B. Wu leg.; XUX-2013-349, 351, 352, 354 to 359.

####### Distribution.

The species is known from the Kagoshima prefecture on the Japanese island Kyushu (Fig. 1C).

###### Heptathela yakushimaensis

Taxon classificationAnimaliaAraneaeLiphistiidae

Ono, 1998

93A7B0B1-F5C7-51FD-AF14-90890EE18AF2

Fig. 8

Heptathela yakushimaensis Ono, 1998: 23 (holotype: female, from Mt. Kunibaidake, Yakushima Island, Kagoshima-ken, Japan, collected by A. Tanikawa on 15 July 1990, deposited in NMNS, examined); Ono 2009: 83.
Heptathela kimurai yakushimaensis : Haupt 2003: 69.

####### Diagnosis.

**Figure 8.**
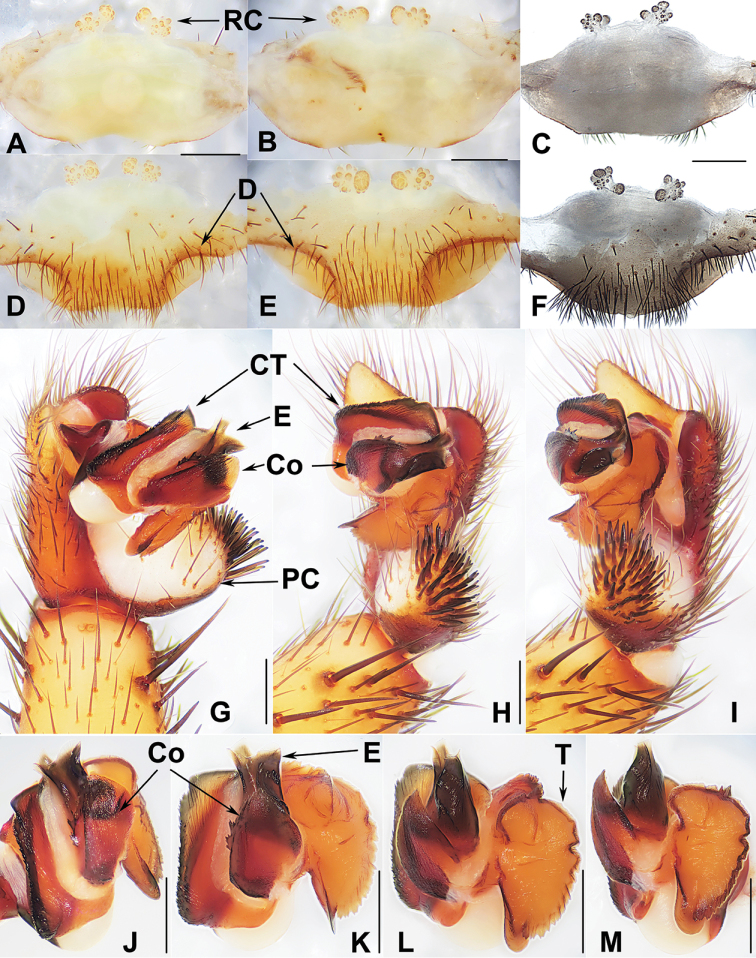
Male and female genital anatomy of *Heptathela yakushimaensis* Ono, 1998 **A, D** 3491 (short for XUX-2013-481) **B, E** 3495 **C, F** 3494 **G–M** 3500 **A–C** vulva dorsal view **D–F** vulva ventral view **G** palp prolateral view **H** palp ventral view **I** palp retrolateral view **J–M** palp distal view. Scale bar: 0.5 mm.

Males of *H. yakushimaensis* differ from those of all other Kyushu group *Heptathela* species by a strongly serrated prolateral conductor margin (Fig. 8G, H, J, K), the tapered tegular marginal apophysis (Fig. 8L, M), and a larger tegular terminal apophysis (Fig. 8L, M). Females of *H. yakushimaensis* differ from those of *H. kimurai* and *H. higoensis* by the finely granulated inner receptacular clusters that are smaller than the outer ones (Fig. 8A–F), and from those of *H. kikuyai* by the inner receptacular clusters lacking well defined stalks. *H. yakushimaensis* also differs from all other Kyushu group *Heptathela* species by the following unique nucleotide substitutions in the standard DNA barcode alignment: T (56), A (68), T (74), G (77), C (84), C (89), C (95), G (98), C (107), A (110), C (122), T (131), T (143), T (164), T (167), C (188), C (200), C (212), T (215), C (216), G (218), T (236), G (242), T (248), G (251), C (278), A (284), T (293), C (294), C (308), T (323), C (347), C (356), T (392), T (395), C (396), T (401), T (407), C (411), C (413), C (417), G (422), G (425), C (437), C (438), A (443), T (455), T (458), T (461), T (482), T (488), C (510), T (528), A (530), A (536), A (539), T (548), G (557), C (567), C (584), T (591), T (632), T (635), G (638), T (650), A (665).

####### Description.

**Males** (*N* = 2). Carapace brown; opisthosoma light brown, with dark brown tergites close to each other; cheliceral groove with 10–13 denticles; 7 spinnerets. Measurements: BL 8.70–10.50, CL 4.27–5.50, CW 4.00–4.90, OL 3.80–6.10, OW 2.40–4.40; ALE > PLE > PME > AME; leg I 13.27 (3.70 + 1.60 + 2.72 + 3.50 + 1.75), leg II 13.71 (3.55 + 1.65 + 2.80 + 3.75 + 1.96), leg III 14.65 (3.52 + 1.65 + 2.78 + 4.50 + 2.20), leg IV 18.64 (4.35 + 1.70 + 3.72 + 6.20 + 2.67).

***Palp*.** Prolateral side of paracymbium unpigmented and unsclerotised, numerous setae and spines at the tip of paracymbium (Fig. 8G–I). Contrategulum with serrated margin proximally and smooth margin distally, and slightly curved at the proximal 2/3 of contrategulum (Fig. 8G, H, J, K). Tegulum with serrated margin, widest in the middle (Fig. 8K–M). Conductor prolateral margin strongly serrated (Fig. 8G, H, J, K). Embolus wide with two peaks (Fig. 8G–K).

**Females** (*N* = 8). Carapace and opisthosoma colour as in male; cheliceral groove with 12–15 pronounced denticles; tergites similar to male; 7 spinnerets. Measurements: BL 9.10–14.80, CL 4.50–6.80, CW 4.05–5.90, OL 4.70–8.60, OW 3.00–6.40; ALE > PLE > PME > AME; palp 9.72 (3.22 + 1.70 + 2.10 + 2.70), leg I 11.15 (3.45 + 1.90 + 2.05 + 2.35 + 1.40), leg II 9.61 (3.07 + 1.90 + 1.00 + 2.36 + 1.28), leg III 11.75 (3.25 + 1.95 + 2.05 + 2.80 + 1.70), leg IV 16.30 (4.25 + 2.25 + 2.90 + 4.40 + 2.50).

***Female genitalia*.** A pair of depressions on the ventro-lateral part of genital atrium. A pair of receptacular clusters along the anterior margin of bursa copulatrix, divided into two parts, the inner part is similar or smaller than the outer part, on which there are several small granules (Fig. 8A–F).

####### Material examined.

JAPAN · 2 ♂♂, 8 ♀♀; Kagoshima-ken, Kumage-gun, Yakushima-cho, Mt. Kankake-dake; 30.37N, 130.39E; alt. 170 m; 24 September 2013; D. Li and B. Wu leg.; XUX-2013-490, 491, 493 to 500 (500, ♂ matured 2 August 2014 at CBEE).

####### Distribution.

The species is known from the Kagoshima prefecture on the Japanese island Yakushima (Fig. 1C).

##### The Amami group

**Figure 9.**
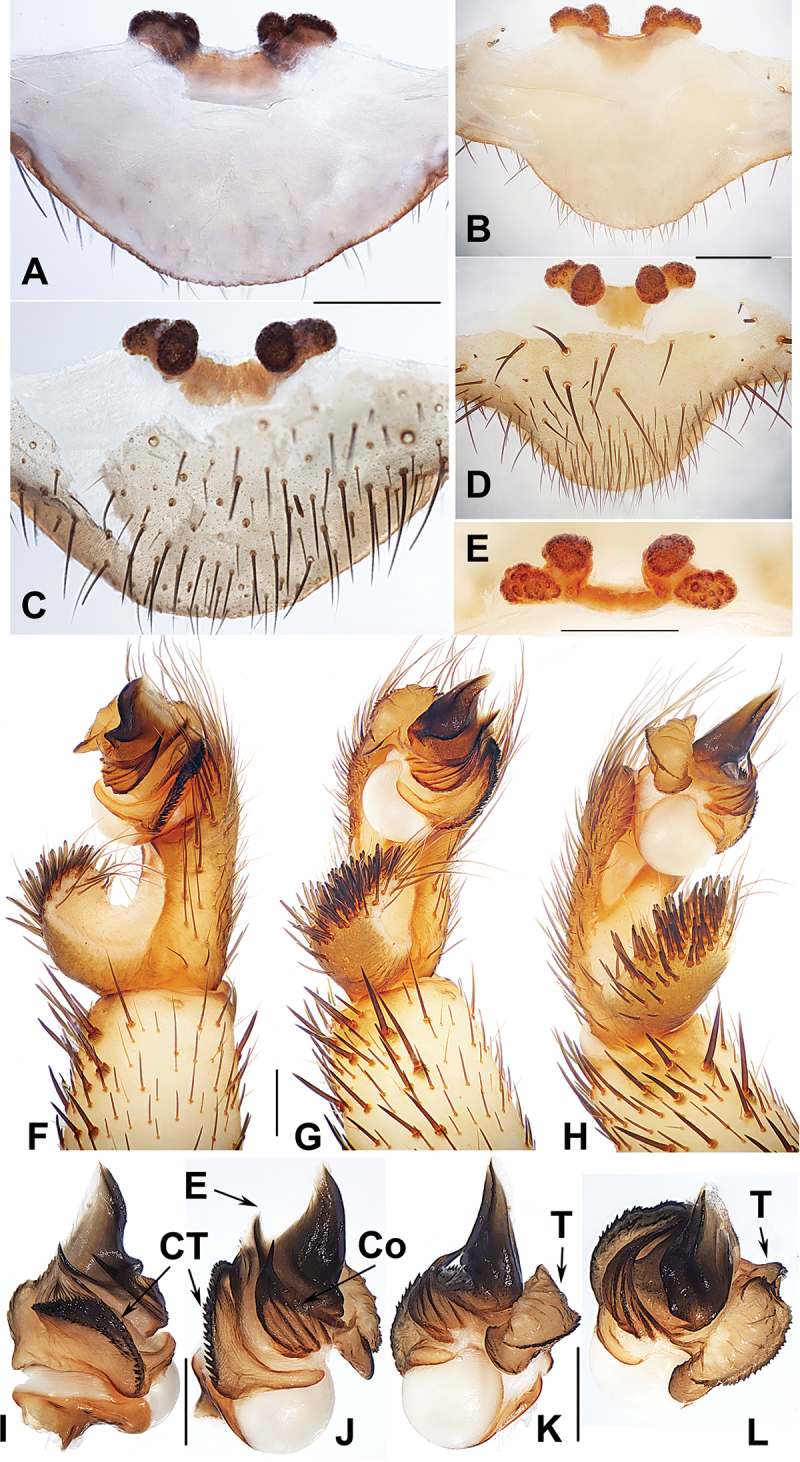
Male and female genital anatomy of *Heptathela amamiensis* Haupt, 1983 **A, C** 3278 (short for XUX-2013-278) **B, D, E** 3285 **F–L** 3283 **A, B** vulva dorsal view **C, D** vulva ventral view **E** vulva distal view **F** right palp prolateral view **G** right palp ventral view **H** right palp retrolateral view **I–L** left palp distal view. Scale bar: 0.5 mm.

**Diagnosis.** The males of the Amami group differ from those of the other two groups by the rugose conductor with a spiniform apex, the contrategulum with a strongly serrated margin whereas it is nearly rectangular in Kyushu group and weakly serrated in Okinawa group, and the embolus with a wide and flat opening (Fig. 9I–L). The females of the Amami group differ from those of the other two groups by indistinctly paired depressions on the ventro-lateral part of the genital atrium and the inner receptacular clusters without tubercula (Fig. 9A–E).

**Monophyly.** The Bayesian analyses based on concatenated two genes and two partitions (for details, see Xu et al. 2019) inferred the same topology, supporting the Amami group monophyly (PP = 1/1) (Fig. 3).

**Composition.***H. amamiensis* Haupt, 1983, *H. kanenoi* Ono, 1996, *H. kojima* sp. nov., *H. sumiyo* sp. nov., and *H. uken* sp. nov.

**Distribution.** Amamioshima and Tokunoshima (Fig. 1C).

###### Heptathela amamiensis

Taxon classificationAnimaliaAraneaeLiphistiidae

Haupt, 1983

2F39360A-6A61-5384-B97F-5C9A29D62EB3

Fig. 9

Heptathela kimurai amamiensis Haupt, 1983: 283 (holotype: female, from Naze, Amami-oshima, Japan, collected by J. Haupt on 26 March 1980, deposited in ZMH, holotype presumably lost (Dunlop et al. 2014); Haupt 2003: 69. Heptathela amamiensis: Ono and Nishikawa 1989: 120; Ono 2009: 80; Ono and Ogata 2018: 27, 479.

####### Diagnosis.

Males of *H. amamiensis* differ from those of *H. sumiyo* sp. nov. by the wider saddle-shaped embolus in the prolateral view, and the narrower conductor base in the ventral view (Fig. 9G, J–L); from those of *H. kanenoi* and *H. kojima* sp. nov. by the spiniform conductor apex (Fig. 9F, I, J). Females of *H. amamiensis* resemble those of other Amami group *Heptathela* species but can be distinguished from those of *H. kanenoi* by the tuberculate outer receptacular clusters (Fig. 9B–E). *H. amamiensis* can also be diagnosed from all other Amami group *Heptathela* species by the following unique nucleotide substitutions in the standard DNA barcode alignment: C (89), A (179), A (194), T (215), T (218), C (273), A (281), C (284), A (327), G (332), G (362), C (467), C (543), C (647).

####### Description.

**Male.** Carapace brown; opisthosoma light brown, with dark brown tergites; cheliceral groove with 13 denticles; 8 spinnerets. Measurements: BL 12.85, CL 6.50, CW 5.98, OL 6.65, OW 4.00; ALE > PLE > PME > AME; leg I 18.25 (5.00 + 2.45 + 3.80 + 4.60 + 2.40), leg II 18.75 (4.85 + 2.40 + 3.75 + 5.00 + 2.75), leg III 20.10 (4.90 + 2.60 + 3.60 + 5.90 + 3.10), leg IV 25.30 (6.00 + 2.60 + 5.10 + 7.70 + 3.90).

***Palp*.** Prolateral side of paracymbium unpigmented and unsclerotised, numerous setae and spines at the tip of paracymbium (Fig. 9F–H). Contrategulum with serrated margin (Fig. 9F, G, I, J). Tegulum wide, with dentate dorsal extension of terminal apophysis (Fig. 9H, L) and blunt terminal apophysis (Fig. 9H, K, L). Conductor sclerotised and rugose, with several folds and a spiniform apex (Fig. 9F–L). Embolus sclerotised, with a wide and flat opening, the distal part slightly sclerotised, and saddle-shaped in the prolateral view (Fig. 9F–L).

**Females** (*N* = 5). Carapace and opisthosoma colour as in male; cheliceral groove with 12–14 pronounced denticles; tergites similar to those of male; seven or eight spinnerets. Measurements: BL 11.08–16.90, CL 5.45–6.90, CW 4.70–6.20, OL 6.61–9.10, OW 4.70–7.38; ALE > PLE > PME > AME; palp 12.36 (4.12 + 2.11 + 2.75 + 3.38), leg I 14.12 (4.52 + 2.50 + 2.70 + 2.83 + 1.57), leg II 14.06 (4.25 + 2.37 + 2.61 + 3.15 + 1.68), leg III 15.13 (4.27 + 2.56 + 2.55 + 3.55 + 2.20), leg IV 21.86 (5.84 + 2.91 + 4.08 + 5.81 + 3.22).

***Female genitalia*.** A pair of indistinct depressions on the ventro-lateral part of genital atrium (Fig. 9C, D). Paired receptacular clusters along the anterior margin of bursa copulatrix, divided into two parts, the inners ovate, the outers tuberculate, without genital stalks (Fig. 9A-E).

####### Material examined.

JAPAN · 2 ♂♂, 5 ♀♀; Kagoshima-ken, Amami-Oshima, Amami-shi, Nazehirata-cho, Michinoshima Loop Bridge; 28.36N, 129.50E; alt. 60 m; 15 September 2013; D. Li and B. Wu leg.; XUX-2013-276, 278, 281 to 285.

####### Distribution.

The species is known from the Japanese island Amamioshima (Fig. 1C).

###### Heptathela kanenoi

Taxon classificationAnimaliaAraneaeLiphistiidae

Ono, 1996

E5CED3E8-05D8-5B31-8E13-363C780C18CC

Fig. 10

Heptathela kanenoi Ono, 1996: 158 (holotype: male, from Mikyo, Amagi-cho, Tokunoshima, Kagoshima-ken, Japan, collected by M. Owada and S. Kaneno on 2 November 1992, deposited in NMNS, examined); Ono 2009: 80.

####### Diagnosis.

**Figure 10.**
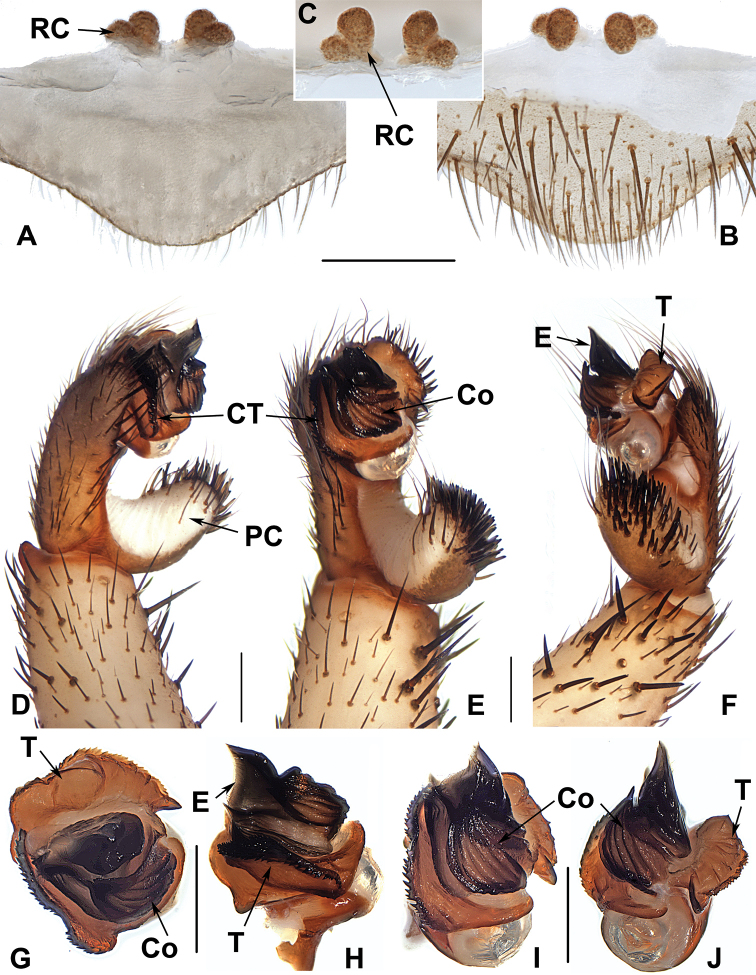
Male and female genital anatomy of *Heptathela kanenoi* Ono, 1996 **A–C** 3338 (short for XUX-2013-338) **D–J** 3334 **A** vulva dorsal view **B** vulva ventral view **C** vulva distal view **D** palp prolateral view **E** palp ventral view **F** palp retrolateral view **G–J** palp distal view. Scale bar: 0.5 mm.

Males of *H. kanenoi* can be distinguished from those of all other Amami group *Heptathela* species by lacking a spiniform conductor apex (Fig. 10G, I). Females of *H. kanenoi* can be distinguished from those of all other Amami group *Heptathela* species by the inner receptacular clusters larger than the outers (Fig. 10B, C). *H. kanenoi* can also be diagnosed from all other Amami group *Heptathela* species by the following unique nucleotide substitutions in the standard DNA barcode alignment: A (35), G (38), C (71), C (125), C (224), G (278), T (281), C (288), T (332), G (359), C (396), C (410), G (443), T (449), A (512), C (533), G (557), C (560), C (623), T (641).

####### Description.

**Males** (*N* = 6). Carapace yellow brown; opisthosoma brown, with dark-spotted tergites close to each other; cheliceral groove with 10–13 vestigial denticles; 7 spinnerets. Measurements: BL 9.80–11.60, CL 5.10–6.00, CW 4.60–5.40, OL 5.15–5.60, OW 4.00–4.85; ALE > PLE > PME > AME; leg I 17.85 (4.80 + 2.30 + 3.80 + 4.60 + 2.35), leg II 18.90 (4.90 + 2.30 + 3.80 + 5.20 + 2.70), leg III 20.80 (4.70 + 2.30 + 4.00 + 6.50 + 3.30), leg IV 26.00 (6.20 + 2.50 + 5.10 + 8.20 + 4.00).

***Palp*.** Prolateral side of paracymbium unpigmented and unsclerotised, numerous setae and spines at the tip of paracymbium (Fig. 10D–F). Contrategulum with serrated margin (Fig. 10G–I). Tegulum with dentate dorsal extension of terminal apophysis (Fig. 10J) and blunt tegulum terminal apophysis (Fig. 10I, J). Conductor base wide and rugose, with several folds and each fold with an apical tooth (Fig. 10G–J). Embolus with a wide and flat opening, the distal part slightly sclerotised, and saddle-shaped in the prolateral view (Fig. 10H–I).

**Females** (*N* = 11). Carapace and opisthosoma colour as in male; cheliceral groove with 12–14 pronounced denticles; tergites similar to male; seven or eight spinnerets. Measurements: BL 8.30–12.90, CL 4.30–6.28, CW 3.60–5.40, OL 4.38–6.50, OW 3.70–6.20; ALE > PLE > PME > AME; palp 7.00 (2.50 + 1.35 + 1.55 + 1.60), leg I 7.75 (2.30 + 1.25 + 1.60 + 1.50 + 1.10), leg II 7.60 (2.60 + 1.10 + 1.50 + 1.50 + 0.90), leg III 8.35 (2.45 + 1.50 + 1.10 + 2.00 + 1.30), leg IV 12.15 (3.40 + 1.50 + 2.25 + 3.20 + 1.80).

***Female genitalia*.** A pair of indistinct depressions on the ventro-lateral part of genital atrium. Paired receptacular clusters along the anterior margin of bursa copulatrix, divided into two parts, the inners larger than the outers, without genital stalks (Fig. 10A–C).

####### Material examined.

JAPAN · 1 ♂, 1 ♀; Kagoshima-ken, Tokunoshima, Amagi-cho, Mikyo; 27.77N, 128.95E; alt. 180 m; 16 September 2013; D. Li and B. Wu leg.; XUX-2013-315 to 316 · 3 ♂♂, 7 ♀♀; Kagoshima-ken, Oshima-gun, Tokunoshima, Tokunoshima-cho, Tokuwase; 27.79N, 129.01E; alt. 150 m; 17 September 2013; D. Li and B. Wu leg.; XUX-2013-323 to 332 · 2 ♂♂, 3 ♀♀; Kagoshima-ken, Tokunoshima, Amagi-cho, Mikyo; 27.77N, 128.95E; alt. 130 m; 17 September 2013; D. Li and B. Wu leg.; XUX-2013-333 to 338.

####### Distribution.

The species is endemic to the Japanese island Tokunoshima (Fig. 1C).

###### Heptathela kojima sp. nov.

Taxon classificationAnimaliaAraneaeLiphistiidae

5D1DC7E6-47F6-50DA-A232-982BF0B8919B

http://zoobank.org/9330F719-A1B4-4169-9FBF-90D13CD49248

Fig. 11

####### Type material.

***Holotype***: JAPAN · ♂; Kagoshima-ken, Oshima-gun, Tokunoshima, Isen-cho, Kojima; 27.74N, 128.91E; alt. 160 m; 17 September 2013; D. Li and B. Wu leg.; XUX-2013-346 (matured 10 October 2013 at CBEE).

***Paratypes***: JAPAN · 2 ♂♂, 6 ♀♀; same data as for holotype; XUX-2013-339, 340, 342 to 345, 347, 348.

####### Diagnosis.

**Figure 11.**
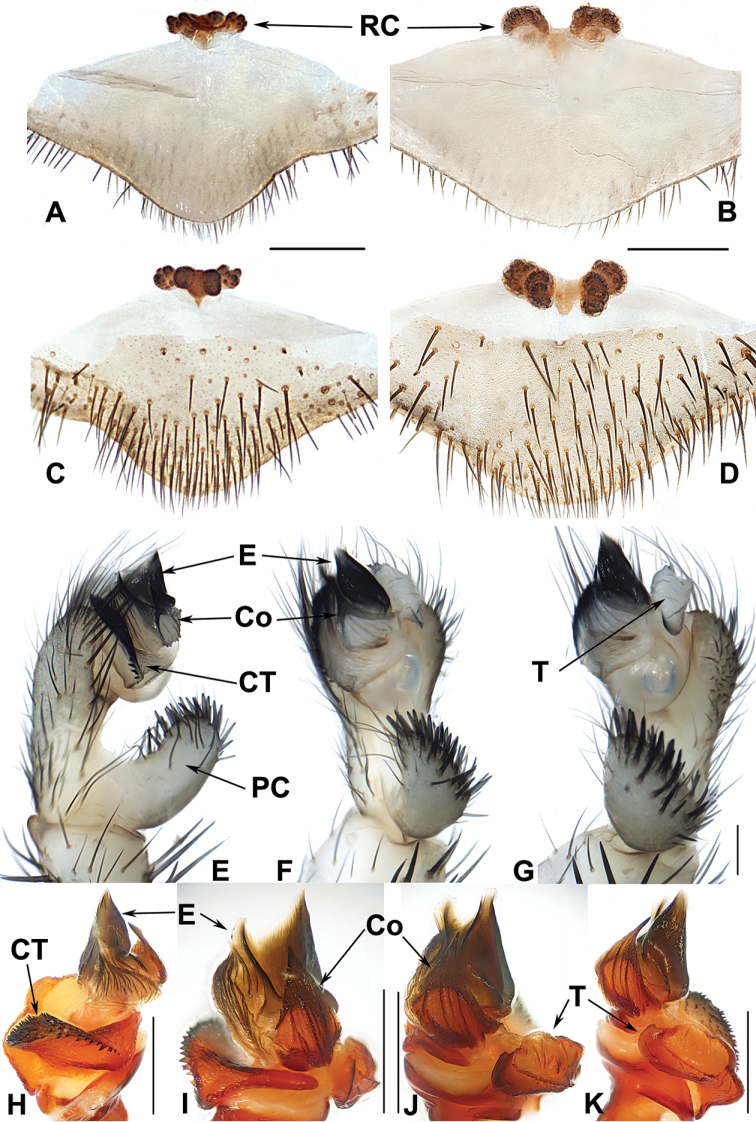
Male and female genital anatomy of *Heptathela kojima* sp. nov. **A, C** 3339 (short for XUX-2013-339) **B, D** 3340 **E–G** 3346 (holotype) **H–K** 3344 **A, B** vulva dorsal view **C, D** vulva ventral view **E** palp prolateral view **F** palp ventral view **G** palp retrolateral view **H–K** palp distal view. Scale bar: 0.5 mm.

Males of *H. kojima* sp. nov. differ from those of *H. amamiensis* and *H. kanenoi* by a wide leaf-shaped conductor (Fig. 11I, J), and a less dentate dorsal extension of the tegular terminal apophysis (Fig. 11G, J, K), from those of *H. sumiyo* sp. nov. by a shallow saddle-shaped in the prolateral view, and from those of *H. uken* sp. nov. by embolus with two longer peaks (Fig. 11I, J). Females of *H. kojima* sp. nov. resemble those of other Amami group *Heptathela* species but differ from those of other Amami group *Heptathela* species by paired receptacular clusters close to each other (Fig. 11B, D). *H. kojima* sp. nov. can also be diagnosed from all other Amami group *Heptathela* species by the following unique nucleotide substitutions in the standard DNA barcode alignment: C (44), C (56), C (128), A (131), C (134), C (137), C (155), G (158), G (176), T (230), T (245), C (269), T (320), C (357), C (377), A (378), A (443), C (446), G (464), A (479), C (518), G (521), T (554), A (560), C (608), C (611).

####### Description.

**Male** (Holotype). Carapace brown; opisthosoma light brown, with dark brown tergites close to each other; cheliceral groove with eleven denticles; seven spinnerets. Measurements: BL 7.30, CL 3.60, CW 3.30, OL 3.60, OW 2.70; ALE > PLE > PME > AME; leg I 14.90 (4.20 + 165 + 3.15 + 4.00 + 1.90), leg II 16.10 (4.20 + 1.85 + 3.25 + 4.40 + 2.40), leg III 16.85 (4.25 + 1.90 + 3.20 + 5.00 + 2.50), leg IV 21.70 (5.50 + 2.00 + 4.20 + 6.90 + 3.10).

***Palp*.** Prolateral side of paracymbium unpigmented and unsclerotised, numerous setae and spines at the tip of paracymbium (Fig. 11E–G). Contrategulum with serrated margin (Fig. 11E, H, I). Conductor base wide, leaf-shaped and rugose, with several folds and gradually narrowing to a short spiniform apex (Fig. 11I, J). Embolus sclerotised, with a wide and flat opening (Fig. 11H–K).

**Females** (*N* = 6). Carapace and opisthosoma colour as in male; cheliceral groove with 13 pronounced denticles; tergites similar to male; 6–8 spinnerets. Measurements: BL 9.00–11.50, CL 4.90–6.15, CW 4.25–5.20, OL 4.30–6.10, OW 3.00–4.50; ALE > PLE > PME > AME; palp 9.05 (3.25 + 1.65 + 1.85 + 2.30), leg I 10.50 (3.35 + 1.80 + 2.00 + 2.10 + 1.25), leg II 10.05 (3.10 + 1.80 + 1.75 + 2.10 + 1.30), leg III 10.45 (2.90 + 1.95 + 1.65 + 2.40 + 1.55), leg IV 15.20 (4.40 + 2.20 + 2.60 + 4.00 + 2.00).

***Female genitalia*.** A pair of indistinct depressions on the ventro-lateral part of genital atrium (Fig. 11C, D). Paired receptacular clusters separated from each other along the anterior margin of bursa copulatrix, or fused together, divided into two parts, without genital stalks (Fig. 11A–D).

####### Etymology.

The species epithet, a noun in apposition, refers to the type locality.

####### Distribution.

The species is endemic to the Japanese island Tokunoshima (Fig. 1C).

###### Heptathela sumiyo sp. nov.

Taxon classificationAnimaliaAraneaeLiphistiidae

4844BEF0-D3BC-5B0D-B6ED-461817EBBFBA

http://zoobank.org/BB1A7494-C5CD-4D69-BC49-67052BCF7E05

Fig. 12

####### Type material.

***Holotype***: JAPAN · ♂; Kagoshima-ken, Amami-Oshima, Amami-shi, Sumiyo-cho, Santaro-toge Pass; 28.28N, 129.42E; alt. 360 m; 15 September 2013; D. Li and B. Wu leg.; XUX-2013-293.

***Paratypes***: JAPAN · 4 ♂♂, 6 ♀♀; same data as for holotype; XUX-2013-287 to 292, 294 to 296B.

####### Diagnosis.

**Figure 12.**
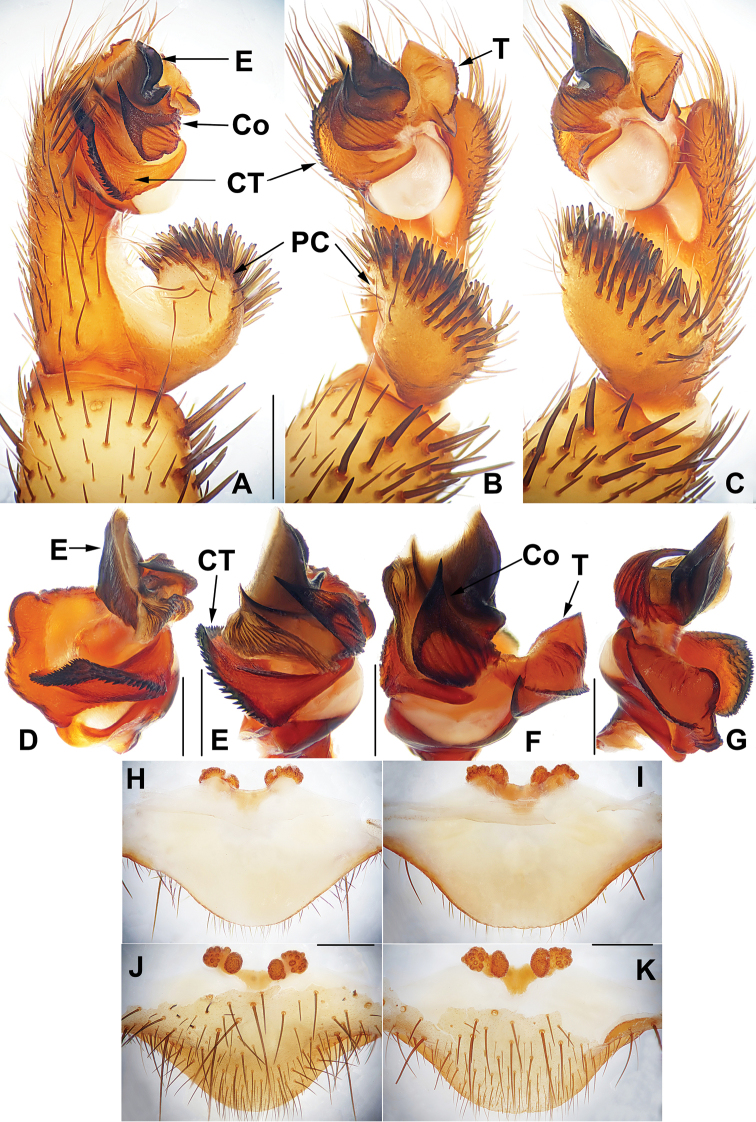
Male and female genital anatomy of *Heptathela sumiyo* sp. nov. **A–C** 3293 (holotype, short for XUX-2013-293) **D–G** 3292 **H, J** 3288 **I, K** 3296 **A** palp prolateral view **B** palp ventral view **C** palp retrolateral view **D–G** palp distal view **H, I** vulva dorsal view **J, K** vulva ventral view. Scale bar: 0.5 mm.

Males of *H. sumiyo* sp. nov. can be distinguished from those of *H. kanenoi* by the spiniform conductor apex (Fig. 12A, E, F), from those of *H. amamiensis* by the narrow and deeper saddle-shaped embolus in the prolateral view (Fig. 12A, E, F). Females of *H. sumiyo* sp. nov. resemble those of the other Amami group *Heptathela* species but differ from those of *H. kanenoi* by the tuberculate lateral receptacular clusters that are equal in size, or slightly larger than the inner clusters (Fig. 12J, K). *H. sumiyo* sp. nov. also differs from all other *Heptathela* species of the Amami group by the following unique nucleotide substitutions in the standard DNA barcode alignment: G (92), C (218), A (227), G (281), C (308), A (363), T (647).

####### Description.

**Male** (Holotype). Carapace brown; opisthosoma light brown, with dark brown tergites; cheliceral groove with eleven denticles; seven spinnerets. Measurements: BL 15.40, CL 7.70, CW 6.80, OL 8.00, OW 5.55; ALE > PLE > PME > AME; leg I 23.05 (6.05 + 3.10 + 4.90 + 6.10 + 2.90), leg II 24.46 (6.48 + 3.08 + 5.05 + 6.80 + 3.05), leg III 26.36 (6.10 + 3.20 + 5.05 + 8.20 + 3.81), leg IV 32.70 (8.00 + 3.50 + 6.75 + 9.70 + 4.75).

***Palp*.** Prolateral side of paracymbium unpigmented and unsclerotised, numerous setae and spines at the tip of paracymbium (Fig. 12A–C). Contrategulum with serrated margin (Fig. 12A, D, E). Tegulum wide with dentate dorsal extension of terminal apophysis (Fig. 12F, G) and blunt terminal apophysis (Fig. 12B, C, F, G). Conductor sclerotised and rugose, with several folds and a spiniform apex (Fig. 12A–B, E–F). Embolus largely sclerotised, with a wide and flat opening, the distal part slightly sclerotised, and narrow and deep saddle-shaped in the prolateral view (Fig. 12A, D–F).

**Females** (*N* = 6). Carapace and opisthosoma colour as in male; cheliceral groove with 13 or 14 pronounced denticles; tergites similar to those of male; seven or eight spinnerets. Measurements: BL 11.70–15.60, CL 6.25–7.55, CW 5.40–6.43, OL 5.40–8.80, OW 4.00–7.35; ALE > PLE > PME > AME; palp 12.90 (4.50 + 2.25 + 2.70 + 3.45), leg I 14.44 (4.55 + 2.68 + 2.55 + 3.08 + 1.58), leg II 13.93 (4.09 + 2.49 + 2.35 + 3.20 + 1.80), leg III 15.61 (4.40 + 2.70 + 2.68 + 3.80 + 2.03), leg IV 22.45 (6.20 + 3.20 + 4.15 + 6.10 + 2.80).

***Female genitalia*.** A pair of indistinct depressions on the ventro-lateral part of genital atrium (Fig. 12J, K). Two pairs of receptacular cluster along the anterior margin of bursa copulatrix, the medians ovate, the laterals tuberculate, similar or slightly larger than inners, without genital stalks (Fig. 12H–K).

####### Etymology.

The species epithet, a noun in apposition, refers to the type locality.

####### Distribution.

The species is known from the Japanese island Amamioshima (Fig. 1C).

###### Heptathela uken sp. nov.

Taxon classificationAnimaliaAraneaeLiphistiidae

7F077D2B-A4FC-5DCC-95C6-D09E666966EF

http://zoobank.org/C538969C-60CB-4184-B388-E8958807AF33

Fig. 13

####### Type material.

***Holotype***: JAPAN · ♂; Kagoshima-ken, Amami, Uken-son, Oshima-gun, Road No. 85, Redsoil Park; 28.24N, 129.34E; alt. 260 m; 15 September 2013; D. Li and B. Wu leg.; XUX-2013-297.

***Paratypes***: JAPAN · 2 ♂♂, 2 ♀♀; same data as for holotype; XUX-2013-298, 301, 302, 304 · 3 ♂♂, 5 ♀♀; Kagoshima-ken, Amami, Yamato-son, Amami Forest Park; 28.31N, 129.33E; alt. 300 m; 17 September 2013; D. Li and B. Wu leg.; XUX-2013-305 to 314.

####### Diagnosis.

**Figure 13.**
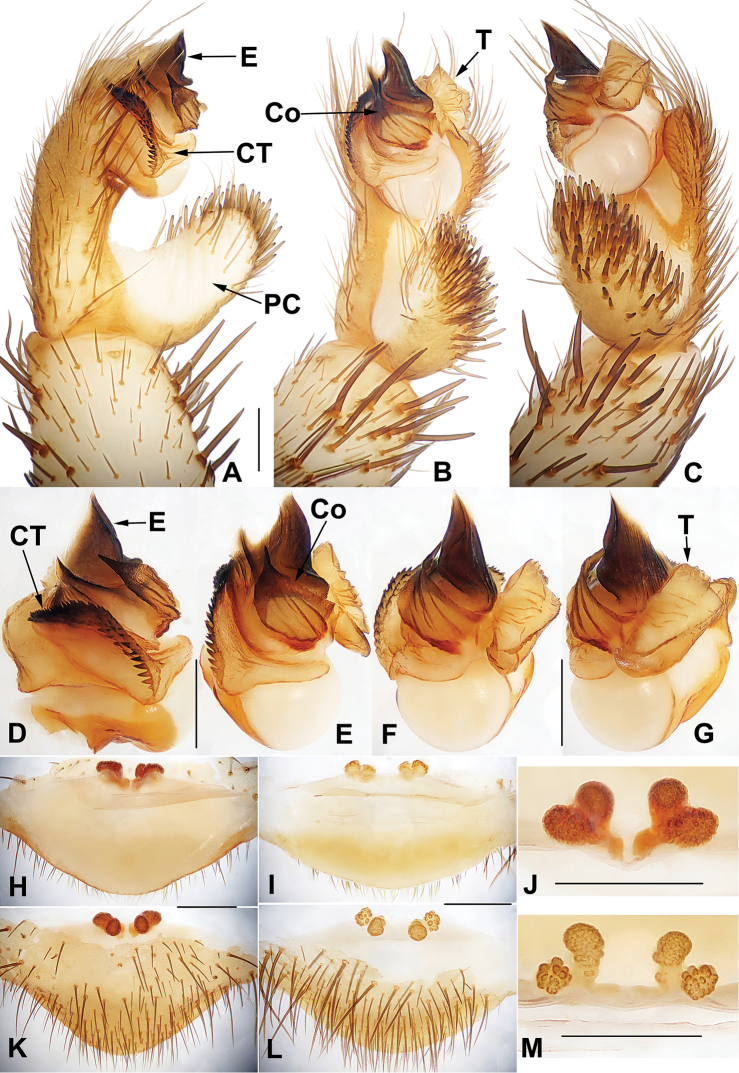
Male and female genital anatomy of *Heptathela uken* sp. nov. **A–G** 3297 (holotype, short for XUX-2013-297) **H, K, J** 3301 **I, L, M** 3309 **A** palp prolateral view **B** palp ventral view **C** palp retrolateral view **D–G** palp distal view **H, I** vulva dorsal view **K, L** vulva ventral view **J, M** vulva distal view; 3297, 3301: Uken-son, Amamioshima; 3309: Yamato-son, Amamioshima. Scale bar: 0.5 mm.

Males of *H. uken* sp. nov. can be distinguished from those of *H. kanenoi* by the spiniform conductor apex (Fig. 13A, B, D, E), from those of *H. amamiensis* by the dorsal extension of tegular terminal apophysis without dentation (Fig. 13F, G). Females of *H. uken* sp. nov. cannot be diagnosed from those of the other Amami group *Heptathela* species morphologically (Fig. 13H–M), only by the following unique nucleotide substitutions in the standard DNA barcode alignment: T (161), T (191), G (227), C (236), T (287), T (297), A (299), C (389), T (395), G (413), C (416), G (503), C (509), C (510), T (527), T (558), G (560), G (569), C (578), T (584), C (596), T (614), G (629), G (635), C (650), C (665).

####### Description.

**Male** (Holotype). Carapace brown; opisthosoma light brown, with dark brown tergites; cheliceral groove with eight denticles; seven spinnerets. Measurements: BL 11.60, CL 6.10, CW 5.60, OL 6.00, OW 4.30; ALE > PLE > PME > AME; leg I 17.47 (4.80 + 2.28 + 3.67+ 4.42 + 2.30), leg II 17.95 (4.65 + 2.30 + 3.60 + 4.90 + 2.50), leg III 18.80 (4.45 + 2.40 + 3.55 + 5.20 + 3.20), leg IV 23.60 (5.60 + 2.45 + 4.45 + 7.20 + 3.90).

***Palp*.** Prolateral side of paracymbium unpigmented and unsclerotised, numerous setae and spines at the tip of paracymbium (Fig. 13A–C). Contrategulum with serrated margin (Fig. 13A, B, D, F). Tegulum with smooth dorsal extension of terminal apophysis (Fig. 13F, G). Conductor wide, sclerotised and rugose, with several folds and a spiniform apex (Fig. 13A, B, D, F). Embolus sclerotised, wide with a flat opening, and wide saddle-shaped in the prolateral view (Fig. 13A, B, D, E).

**Females** (*N* = 8). Carapace and opisthosoma colour as in male; cheliceral groove with 13–15 pronounced denticles; tergites similar to male; 7 or 8 spinnerets. Measurements: BL 11.80–16.00, CL 5.90–8.26, CW 5.20–7.20, OL 6.00–8.10, OW 4.60–6.00; ALE > PLE > PME > AME; palp 10.04 (3.05 + 1.89 + 2.30 + 2.80), leg I 12.05 (3.90 + 2.10 + 2.25 + 2.40 + 1.40), leg II 11.30 (3.55 + 2.05 + 1.80+ 2.45 + 1.45), leg III 12.42 (3.60 + 2.05 + 2.10 + 3.00 + 1.67), leg IV 18.33 (5.15 + 2.40 + 3.30 + 4.78 + 2.70).

***Female genitalia*.** A pair of indistinct depressions on the ventro-lateral part of genital atrium (Fig. 13K). Two pairs of receptacular clusters along the anterior margin of bursa copulatrix, the inners almost globose, with short genital stalks in distal view, the laterals tuberculate, without genital stalks (Fig. 13H–M).

####### Etymology.

The species epithet, a noun in apposition, refers to the type locality.

####### Distribution.

The species is known from the Japanese island Amamioshima (Fig. 1C).

##### The Okinawa group

**Figure 14.**
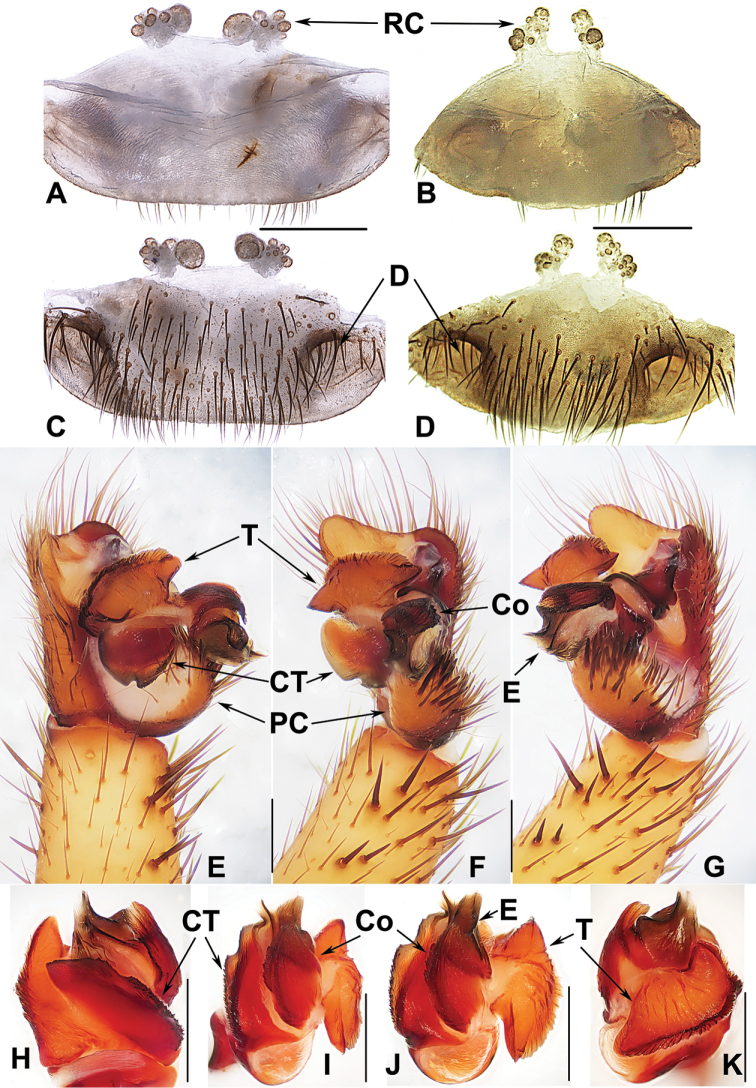
Male and female genital anatomy of *Heptathela yanbaruensis* Haupt, 1983 **A, C** 2315 (short for XUX-2012-315) **B, D** 2441 **E–G** 2322B **H–K** 2311 **A, B** vulva dorsal view **C, D** vulva ventral view **E** palp prolateral view **F** palp ventral view **G** palp retrolateral view **H–K** palp distal view; 2311, 2315, 2322B: Yona, Okinawajima; 2441: Taiho Dam, Okinawajima. Scale bar: 0.5 mm.

**Diagnosis.** The males of the Okinawa group differ from those of the Kyushu group by the semi-elliptic contrategulum (Fig. 14H–J), and from those of the Amami group by the contrategulum whose margin is only weakly serrated, is curved in the middle, and is proximally serrated and distally smooth (Fig. 14I, J). The females of the Okinawa group resemble those of the Kyushu group, but differ from those of the Amami group by distinctly paired depressions on the ventro-lateral part of the genital atrium (Fig. 14C, D).

**Monophyly.** The Bayesian analyses based on concatenated two genes and two partitions (for details, see Xu et al. 2019) suggested the Okinawa clades are paraphyly (Fig. 3). Therefore, in this study, Okinawa group includes all species from Okinawa except *H. helios*, which is sister to all the other *Heptathela* species (Fig. 3).

**Composition.***H. yanbaruensis* Haupt, 1983, *H. aha* sp. nov., *H. gayozan* sp. nov., *H. kubayama* sp. nov., *H. mae* sp. nov., *H. otoha* sp. nov., *H. shuri* sp. nov., *H. tokashiki* sp. nov., *H. unten* sp. nov., and *H. crypta* sp. nov.

**Distribution.** Okinawajima, Iheyajima, Tokashikijima (Fig. 1C).

###### Heptathela yanbaruensis

Taxon classificationAnimaliaAraneaeLiphistiidae

Haupt, 1983

F2DC85B7-FA1B-59F2-84FE-30521690E460

Fig. 14

Heptathela kimurai yanbaruensis Haupt, 1983: 284 (holotype: male, from Yona, Okinawa, Japan, collected by J. Haupt on 15 April 1977, deposited in ZMH, where the type may be lost (Dunlop et al. 2014); Haupt 1984: 166; Haupt 2003: 69. Heptathela yanbaruensis: Ono 2009: 80; Ono and Ogata 2018: 28, 480.

####### Diagnosis.

Males of *H. yanbaruensis* can be distinguished from those of *H. helios* by the contrategulum that is distinctly curved in the middle (Fig. 14I, J), and from those of *H. unten* sp. nov. and *H. crypta* sp. nov. by the conductor that is longer than its width (Fig. 14I, J). Females of *H. yanbaruensis* can be distinguished from those of *H. shuri* sp. nov. by the wide and straight posterior margin of the genital atrium (Fig. 14A, C). *Heptathela yanbaruensis* can also be diagnosed from all other *Heptathela* species of the Okinawa group by the following unique nucleotide substitutions in the standard DNA barcode alignment: G (53), T (327), A (356), A (443).

####### Description.

**Males** (*N* = 3). Carapace yellow brown; opisthosoma light brown, with dark-spotted tergites close to each other; cheliceral groove with 10–12 denticles; seven spinnerets. Measurements: BL 8.53–9.22, CL 4.21–4.57, CW 3.71–3.75, OL 4.20–4.48, OW 2.30–2.80; ALE > PLE > PME > AME; leg I 14.28 (4.15 + 1.70 + 3.05 + 3.56 + 1.82), leg II 15.15 (3.97 + 1.70 + 3.07 + 4.13 + 2.28), leg III 15.24 (4.00 + 1.68 + 3.08 + 4.10 + 2.38), leg IV 19.78 (4.75 + 1.37 + 3.91 + 6.45 + 3.30).

***Palp*.** Prolateral side of paracymbium unpigmented and unsclerotised, numerous setae and spines at the tip of paracymbium (Fig. 14E–G). Contrategulum margin obviously curved in the middle, the contrategulum margin proximally serrated and distally smooth (Fig. 14H–J). Tegulum wide with a dentate dorsal extension of terminal apophysis (Fig. 14J, K), blunt terminal and marginal apophysis (Fig. 14J, K). Conductor oval, with weakly serrated margin, and a fold in prolateral view (Fig. 14H–J). Embolus sclerotised, with a wide opening, the distal margin slightly sclerotised, and with a wide saddle-shaped margin in the retrolateral view (Fig. 14I–K).

**Females** (*N* = 15). Carapace and opisthosoma colour as in male; cheliceral groove with 11–14 pronounced denticles; tergites similar to male; seven spinnerets. Measurements: BL 7.78–10.33, CL 3.90–5.10, CW 3.40–4.23, OL 3.90–6.00, OW 2.80–4.70; ALE > PLE > PME > AME; palp 8.70 (2.96 + 1.51 + 2.11 + 2.12), leg I 10.20 (3.12 + 1.75 + 1.93 + 2.12 + 1.28), leg II 9.97 (3.05 + 1.72 + 1.77 + 2.13 + 1.30), leg III 10.05 (2.91 + 1.78 + 1.48 + 2.47 + 1.41), leg IV 14.77 (4.08 + 2.10 + 2.51 + 3.97 + 2.11).

***Female genitalia*.** A pair of depressions on the ventro-lateral part of the genital atrium (Fig. 14C, D). The posterior margin of genital atrium wide and straight (Fig. 14A–D). Paired receptacular clusters along the anterior margin of bursa copulatrix, divided into two parts, the size of inner ones similar to that of laterals, with several granules, with short genital stalks (Fig. 14A–D).

####### Material examined.

JAPAN · 3 ♂♂, 9 ♀♀; Okinawa-ken, Kunigami-son, Yona, Tropical Biosphere Research Centre field station, University of the Ryukyu; 26.76N, 128.22E; alt. 20 m; 18 December 2012; D. Li, F.X. Liu and X. Xu leg.; XUX-2012-310 to 322B · 6 ♀♀; Okinawa-ken, Ogimi-son, Taiho Dam; 26.65N, 128.16E; alt. 80 m; 24 December 2012; D. Li, F.X. Liu and X. Xu leg.; XUX-2012-441 to 446.

####### Distribution.

The species is endemic to the Japanese island Okinawajima (Fig. 1C).

###### Heptathela aha sp. nov.

Taxon classificationAnimaliaAraneaeLiphistiidae

5549718A-7545-5698-841B-516DFA367939

http://zoobank.org/4F130218-5900-4688-AF57-F018C26E34C8

Fig. 15

####### Type material.

***Holotype***: JAPAN · ♀; Okinawa-ken, Iheyajima Island, Mt. Aha-dake; 27.02N, 127.93E; alt. 10 m; 26 December 2012; D. Li, F.X. Liu and X. Xu leg.; XUX-2012-502.

***Paratypes***: JAPAN · 6 ♀♀; same data as for holotype; XUX-2012-504 to 519.

####### Diagnosis.

**Figure 15.**
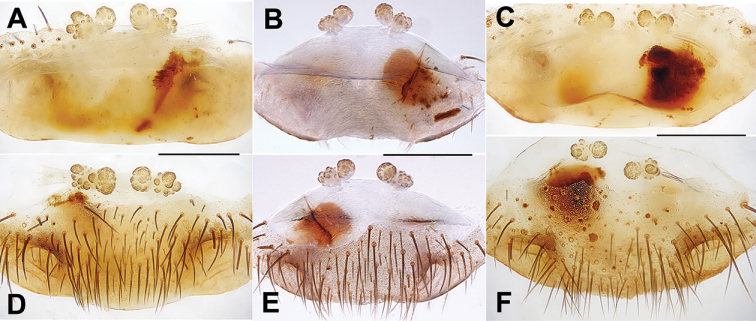
Female genital anatomy of *Heptathela aha* sp. nov. **A, D** 2502 (holotype, short for XUX-2012-502) **B, E** 2505 **C, F** 2506 **A–C** vulva dorsal view **D–F** vulva ventral view. Scale bar: 0.5 mm.

Females of *H. aha* sp. nov. cannot be distinguished morphologically from those of *H. gayozan* sp. nov. but can be distinguished from those of *H. kubayama* sp. nov. by the receptacular clusters without genital stalks; and from those of *H. mae* sp. nov. by the inner receptacular clusters similar to or larger than laterals (Fig. 15A–F). *Heptathela aha* sp. nov. can also be diagnosed from all other Okinawa group *Heptathela* species by the following unique nucleotide substitutions in the standard DNA barcode alignment: C (41), C (179), G (182), G (233), T (248), G (251), T (326), A (347), G (359), C (473), C (492), A (536), T (651).

####### Description.

**Female** (Holotype). Carapace brown; opisthosoma brown, brown tergites with black plaques; cheliceral groove with 12 pronounced denticles; seven spinnerets. Measurements: BL 12.60, CL 5.55, CW 5.00, OL 6.82, OW 5.50; ALE > PLE > PME > AME; palp 9.90 (3.40 + 1.71 + 2.18 + 2.61), leg I 11.34 (3.31 + 2.05 + 2.31 + 2.35 + 1.32), leg II 11.65 (3.51 + 1.98 + 2.03 + 2.53 + 1.60), leg III 11.92 (3.30 + 1.90 + 2.05 + 2.97 + 1.70), leg IV 16.96 (4.88 + 2.22 + 2.98 + 34.45 + 2.43).

***Female genitalia*.** A pair of depressions on the ventro-lateral part of the genital atrium (Fig. 15D–F). A pair of receptacular clusters along the anterior margin of bursa copulatrix, divided into two parts, both without genital stalks (Fig. 15A–F).

**Male.** Unknown.

####### Etymology.

The species epithet, a noun in apposition, refers to the type locality.

####### Distribution.

The species is endemic to the Japanese island Iheyajima (Fig. 1C).

###### Heptathela gayozan sp. nov.

Taxon classificationAnimaliaAraneaeLiphistiidae

20747208-37D4-5BBE-BA64-4BB9719F9647

http://zoobank.org/6DAC9A7B-50DD-4513-ACD6-B51AF264BBC2

Fig. 16

####### Type material.

***Holotype***: JAPAN · ♀; Okinawa-ken, Iheyajima Island, Mt. Gayozan; 27.02N, 127.97E; alt. 25 m; 27 December 2012; D. Li, F.X. Liu and X. Xu leg.; XUX-2012-511.

***Paratypes***: JAPAN · 2 ♀♀; same data as for holotype; XUX-2012-513, 515.

####### Diagnosis.

**Figure 16.**
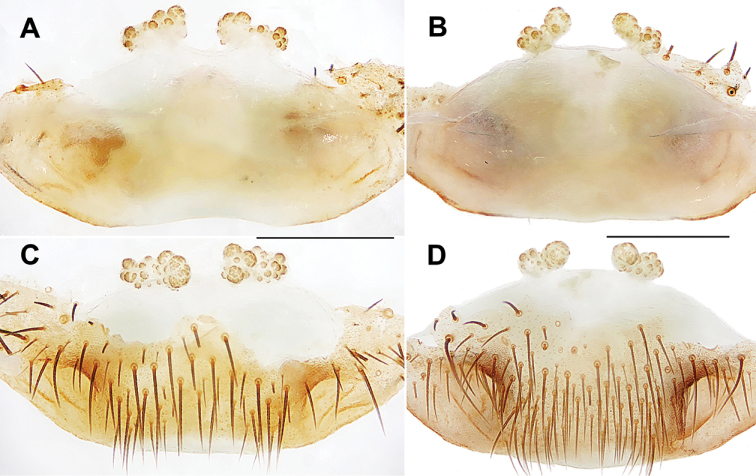
Female genital anatomy of *Heptathela gayozan* sp. nov. **A, C** 2511 (holotype, short for XUX-2012-511) **B, D** 2513 **A, B** vulva dorsal view **C, D** vulva ventral view. Scale bar: 0.5 mm.

Females *H. gayozan* sp. nov. cannot be distinguished morphologically from those of *H. aha* sp. nov. but can be distinguished from those of *H. kubayama* sp. nov. by the receptacular clusters without genital stalks; and from those of *H. mae* sp. nov. by the inner receptacular clusters that are equal in size, or slightly larger than laterals (Fig. 16A–D). *H. gayozan* sp. nov. can be diagnosed from all other Okinawa group *Heptathela* species by the following unique nucleotide substitutions in the standard DNA barcode alignment: G (38), G (41), G (122), C (203), C (365), C (452), C (470), T (518), C (527), C (533), T (560), T (653).

####### Description.

**Female** (holotype). Carapace brown; opisthosoma light brown, with dark brown tergites close to each other; cheliceral groove with 12 denticles; seven spinnerets. Measurements: BL 10.00, CL 4.40, CW 3.88, OL 4.84, OW 3.48; ALE > PLE > PME > AME; palp 7.32 (2.72 + 1.50 + 1.80 + 1.30), leg I 9.61 (2.95 + 1.62 + 1.82 + 1.97 + 1.25), leg II 9.38 (2.80 + 1.58 + 1.71 + 2.07 + 1.22), leg III 9.60 (2.65 + 1.55 + 1.62 + 2.28 + 1.50), leg IV 13.89 (3.75 + 1.82 + 2.52 + 3.75 + 2.05).

***Female genitalia*.** A pair of depressions on the ventro-lateral part of genital atrium (Fig. 16C, D). A pair of receptacular clusters along the anterior margin of bursa copulatrix, divided into two parts, with several granules, without genital stalks (Fig. 16).

**Male.** Unknown.

####### Etymology.

The species epithet, a noun in apposition, refers to the type locality.

####### Distribution.

The species is endemic to the Japanese island Iheyajima (Fig. 1C).

###### Heptathela kubayama sp. nov.

Taxon classificationAnimaliaAraneaeLiphistiidae

2C61BBF7-3765-5063-A5A8-113AC6EA8C44

http://zoobank.org/C8B09629-5191-447F-8C89-9953504F8E0D

Fig. 17

####### Type material.

***Holotype***: JAPAN · ♀; Okinawa-ken, Iheyajima Island, Mt. Kubayama Nature Conservation Area; 27.09N, 128.02E; alt. 85 m; 26 December 2012; D. Li, F.X. Liu and X. Xu leg.; XUX-2012-486.

***Paratypes***: JAPAN · 8 ♀♀; same data as for holotype; XUX-2012-479, 481 to 485, 487 to 488.

####### Diagnosis.

**Figure 17.**
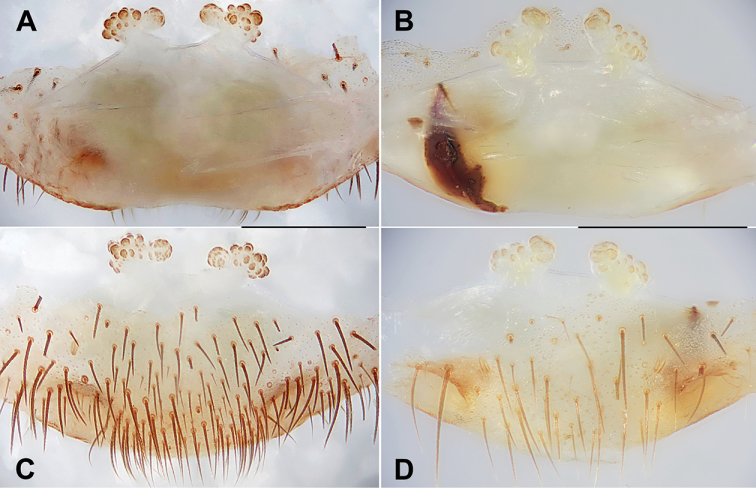
Female genital anatomy of *Heptathela kubayama* sp. nov. **A, C** 2486 (holotype, short for XUX-2012-486) **B, D** 2482 **A, B** vulva dorsal view **C, D** vulva ventral view. Scale bar: 0.5 mm.

Females of *H. kubayama* sp. nov. can be distinguished from those of *H. gayozan* sp. nov. and *H. mae* sp. nov. by paired receptacular clusters with short genital stalks (Fig. 17A–B). *Heptathela kubayama* sp. nov. can also be diagnosed from all other Okinawa group *Heptathela* species by the following unique nucleotide substitutions in the standard DNA barcode alignment: G (239), G (329), G (353), C (359), G (443), G (602), G (647).

####### Description.

**Female** (Holotype). Carapace brown; opisthosoma light brown, with dark brown tergites close to each other; cheliceral groove with 12 pronounced denticles; six spinnerets. Measurements: BL 10.20, CL 4.89, CW 4.18, OL 4.90, OW 3.60; ALE > PLE > PME > AME; palp 8.25 (2.82 + 1.45 + 1.80 + 2.18), leg I 9.66 (3.10 + 1.70 + 1.81 + 1.90 + 1.15), leg II 8.90 (2.90 + 1.55 + 1.75 + 1.70 + 1.00), leg III 9.59 (2.81 + 1.65 + 1.60 + 2.13 + 1.40), leg IV 13.67 (4.25 + 2.00 + 2.00 + 3.54 + 1.88).

***Female genitalia*.** A pair of depressions on the ventro-lateral part of the genital atrium (Fig. 17C, D). A pair of receptacular clusters along the anterior margin of bursa copulatrix, divided into two parts, with several granules; with short genital stalks (Fig. 17A–D).

**Male.** Unknown.

####### Etymology.

The species epithet, a noun in apposition, refers to the type locality.

####### Distribution.

The species is endemic to the Japanese island Iheyajima (Fig. 1C).

###### Heptathela mae sp. nov.

Taxon classificationAnimaliaAraneaeLiphistiidae

99F3A354-2E46-55A0-A8CC-19EACA81F7DA

http://zoobank.org/45944FF0-D6B1-490B-ABCE-8CC5A933EDC3

Fig. 18

####### Type material.

***Holotype***: JAPAN · ♀; Okinawa-ken, Iheyajima Island, Mt. Mae-dake; 27.06N, 127.99E; alt. 10 m; 26 December 2012; D. Li, F.X. Liu and X. Xu leg.; XUX-2012-497.

***Paratypes***: JAPAN · 5 ♀♀; same data as for holotype; XUX-2012-494 to 496, 498, 500 · 3♀♀; Okinawa Prefecture, Iheyajima Island, Mt. Mae-dake; 27.06N, 127.99E; alt. 20 m; 10 May 2014; D. Li and B. Wu leg.; XUX-2014-079A to 079C.

####### Diagnosis.

**Figure 18.**
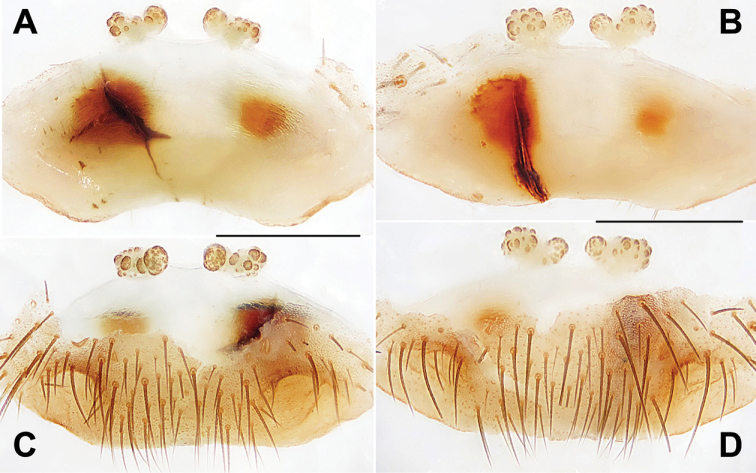
Female genital anatomy of *Heptathela mae* sp. nov. **A, C** 2497 (holotype, short for XUX-2012-497) **B, D** 4079B **A, B** vulva dorsal view **C, D** vulva ventral view. Scale bar: 0.5 mm.

Females of *H. mae* sp. nov. can be distinguished from those of *H. aha* sp. nov. and *H. gayozan* sp. nov. by the lateral receptacular clusters being larger than the inner ones (Fig. 18B, D), and from those of *H. kubayama* sp. nov. by the receptacular clusters without short genital stalks (Fig. 18A–D). *Heptathela mae* sp. nov. can also be diagnosed from all other Okinawa group *Heptathela* species by the following unique nucleotide substitutions in the standard DNA barcode alignment: C (74), T (227), G (389), C (407), A (461), G (503), G (635).

####### Description.

**Female** (Holotype). Carapace brown; opisthosoma brown, brown tergites with black plaques; cheliceral groove with 12 denticles; seven spinnerets. Measurements: BL 9.28, CL 4.48, CW 4.00, OL 5.12, OW 3.78; ALE > PLE > PME > AME; palp 7.80 (2.70 + 1.32 + 1.71 + 2.07), leg I 9.25 (3.00 + 1.52 + 1.80 + 1.78 + 1.15), leg II 8.55 (2.68 + 1.20 + 1.72 + 1.75 + 1.20), leg III 9.61 (2.75 + 1.60 + 1.55 + 2.31 + 1.40), leg IV 13.41 (3.90 + 1.70 + 2.31 + 3.60 + 1.90).

***Female genitalia*.** A pair of depressions on the ventro-lateral part of the genital atrium (Fig. 18C, D). A pair of receptacular clusters along the anterior margin of bursa copulatrix, divided into two parts, with several granules, without genital stalks, the posterior part of genital area incurved (Fig. 18A–D).

**Male.** Unknown.

####### Etymology.

The species epithet, a noun in apposition, refers to the type locality.

####### Distribution.

The species is endemic to the Japanese island Iheyajima (Fig. 1C).

###### Heptathela otoha sp. nov.

Taxon classificationAnimaliaAraneaeLiphistiidae

71A1B990-F843-56DB-AE11-F68F2953B1E2

http://zoobank.org/2C41220D-A64D-4750-9C99-D5F12FB4543E

Fig. 19

####### Type material.

***Holotype***: JAPAN · ♀; Okinawa-ken, Nakijin-son, Mt. Otoha-dake; 26.67N, 127.97E; alt. 80 m; 27 December 2012; D. Li, F.X. Liu and X. Xu leg.; XUX-2012-535.

***Paratypes***: JAPAN · 3 ♀♀; Okinawa Prefecture, Motobu-cho, Yamazato; 26.67N, 127.91E; alt. 160 m; 11 May 2014; D. Li and B. Wu leg.; XUX-2014-094, 097, 099 · 3 ♀♀; Okinawa Prefecture, Nakijin-son, Mt. Otoha-dake; 26.67N, 127.97E; alt. 100 m; 11 May 2014; D. Li and B. Wu leg.; XUX-2014-101, 102, 103.

####### Diagnosis.

**Figure 19.**
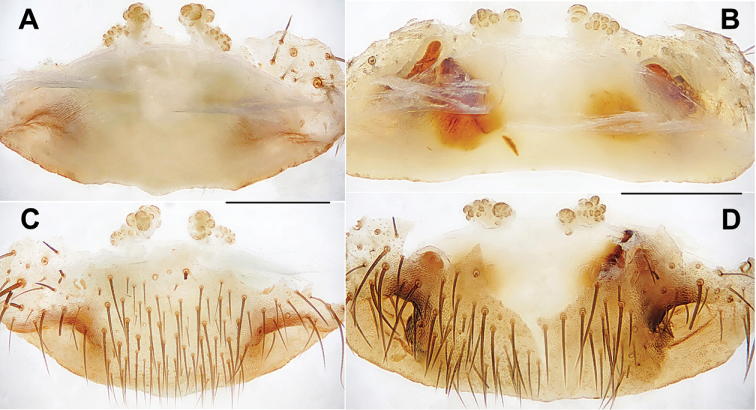
Female genital anatomy of *Heptathela otoha* sp. nov. **A, C** 2535 (holotype, short for XUX-2012-535) **B, D** 4094 **A, B** vulva dorsal view **C, D** vulva ventral view. Scale bar: 0.5 mm.

Females of *H. otoha* sp. nov. can be distinguished from those of *H. yanbaruensis*, *H. unten* sp. nov., and *H. crypta* sp. nov. by the inner receptacular clusters with several granules, albeit these inner clusters are difficult to be separated from the laterals (Fig. 19A–D). *H. otoha* sp. nov. can also be diagnosed from all other Okinawa group *Heptathela* species by the following unique nucleotide substitutions in the standard DNA barcode alignment: G (83), G (206), G (488).

####### Description.

**Female** (Holotype). Carapace yellow brown; opisthosoma light brown, with brown and black-spotted tergites; cheliceral groove with 13 pronounced denticles; seven spinnerets. Measurements: BL 10.92, CL 4.75, CW 4.35, OL 6.60, OW 4.90; ALE > PLE > PME > AME; palp 8.30 (2.78 + 1.39 + 1.90 + 2.23), leg I 9.80 (3.05 + 1.65 + 1.95 + 1.95 + 1.20), leg II 9.21 (2.90 + 1.50 + 1.81 + 1.90 + 1.10), leg III 9.84 (2.70 + 1.70 + 1.62 + 2.35 + 1.47), leg IV 14.74 (4.00 + 1.90 + 2.70 + 4.03 + 2.11).

***Female genitalia*.** A pair of depressions on the ventro-lateral part of genital atrium (Fig. 19C, D). Paired receptacular clusters along the anterior margin of bursa copulatrix, divided into indistinct two parts, but difficulty to separate, both with several granules, with very short genital stalks (Fig. 19A–D).

**Male.** Unknow.

####### Etymology.

The species epithet, a noun in apposition, refers to the type locality.

####### Distribution.

The species is endemic to the Japanese island Okinawajima (Fig. 1C).

###### Heptathela shuri sp. nov.

Taxon classificationAnimaliaAraneaeLiphistiidae

53D77AAC-44A5-548D-B625-D0A92B2BB232

http://zoobank.org/B74BEC88-F046-4583-80F3-E6BD2909BFAC

Fig. 20

####### Type material.

***Holotype***: JAPAN · ♀; Okinawa-ken, Naha, Shuri, Sueyoshi Park; 26.23N, 127.72E; alt. 45 m; 17 December 2012; D. Li, F.X. Liu and X. Xu leg.; XUX-2012-309.

***Paratype***: JAPAN · 1 ♀; same data as for holotype; XUX-2012-308.

####### Diagnosis.

**Figure 20.**
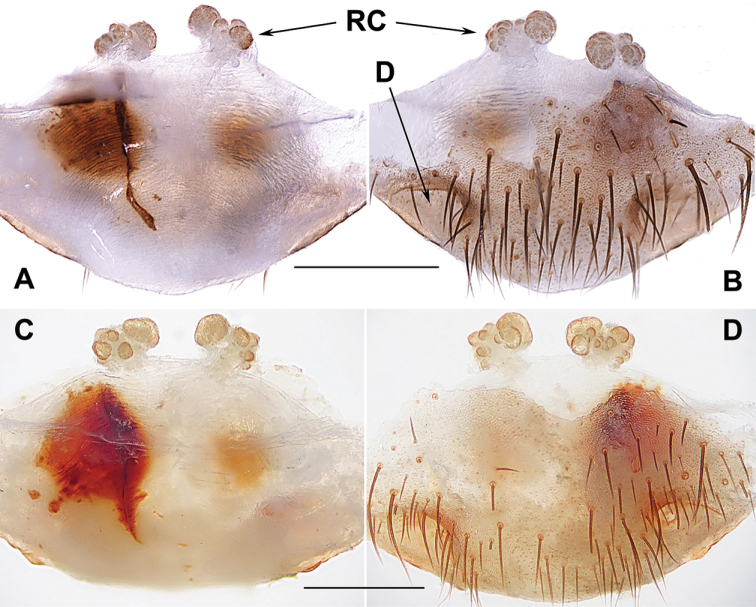
Female genital anatomy of *Heptathela shuri* sp. nov. **A, B** 2308 (short for XUX-2012-308) **C, D** 2309 (holotype) **A, C** vulva dorsal view **B, D** vulva ventral view. Scale bar: 0.5 mm.

Females of *H. shuri* sp. nov. can be distinguished from those of *H. tokashiki* sp. nov. by paired receptacular clusters with larger granules (Fig. 20A–D). *Heptathela shuri* sp. nov. can also be diagnosed from all other Okinawa group *Heptathela* species by the following unique nucleotide substitutions in the standard DNA barcode alignment: G (89), G (131), C (134), C (396), C (510), C (512), A (524), T (641).

####### Description.

**Female** (Holotype). Carapace yellow brown; opisthosoma brown, tergites with brown plaques; cheliceral groove with 12 pronounced denticles; seven spinnerets. Measurements: BL 10.78, CL 5.30, CW 4.12, OL 6.25, OW 4.40; ALE > PLE > PME > AME; palp 8.93 (3.15 + 1.43 + 2.03 + 2.32), leg I 10.91 (3.48 + 1.87 + 2.05 + 2.21 + 1.30), leg II 10.64 (3.18 + 1.85 + 1.86 + 2.28 + 1.47), leg III 11.26 (3.03 + 1.81 + 1.77 + 2.90 + 1.75), leg IV 17.02 (4.62 + 2.28 + 3.07 + 4.50 + 2.55).

***Female genitalia*.** A pair of depressions on the ventro-lateral part of genital atrium (Fig. 20B, D). Paired receptacular clusters along the anterior margin of bursa copulatrix, divided into two parts, with several granules, the inner granules larger than laterals, both without genital stalks (Fig. 20A–D).

**Male.** Unknown.

####### Etymology.

The species epithet, a noun in apposition, refers to the type locality.

####### Distribution.

The species is endemic to the Japanese island Okinawajima (Fig. 1C).

###### Heptathela tokashiki sp. nov.

Taxon classificationAnimaliaAraneaeLiphistiidae

E54E646D-3CE3-518C-9723-B42967CDF3A7

http://zoobank.org/A5A7B2B1-FE6F-4B42-AF06-48E7BDD937CB

Fig. 21

####### Type material.

***Holotype***: JAPAN · ♀; Okinawa-ken, Tokashikijima Island, Aharen; 26.19N, 127.37E; alt. 100 m; 8 May 2014; D. Li and B. Wu leg.; XUX-2014-062.

***Paratypes***: JAPAN · 25 ♀♀; same data as for holotype; XUX-2014-046 to 063D · 3 ♀♀; same data as for holotype; 27 December 2012; D. Li, F.X. Liu and X. Xu leg.; XUX-2012-417, 421, 425.

####### Diagnosis.

**Figure 21.**
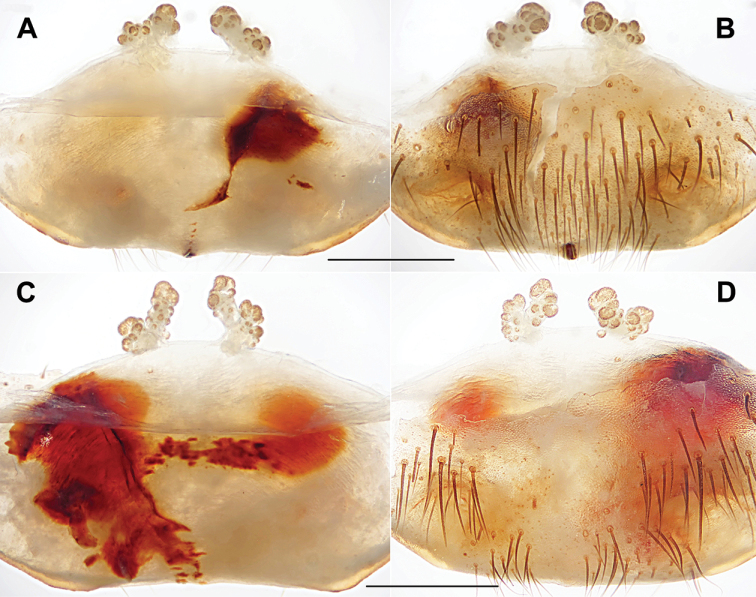
Female genital anatomy of *Heptathela tokashiki* sp. nov. **A, B** 4062 (holotype, short for XUX-2014-062) **C, D** 4063 **A, C** vulva dorsal view **B, D** vulva ventral view. Scale bar: 0.5 mm.

Females of *H. tokashiki* sp. nov. can be distinguished from *H. shuri* sp. nov. by the long inner receptacular clusters with several granules (Fig. 21A–D). *Heptathela tokashiki* sp. nov. can also be diagnosed from all other Okinawa group *Heptathela* species by the following unique nucleotide substitutions in the standard DNA barcode alignment: T (68), A (200), C (290), A (362).

####### Description.

**Female** (Holotype). Carapace brown; opisthosoma dark brown, with dark brown tergites close to each other; cheliceral groove with eleven pronounced denticles; seven spinnerets. Measurements: BL 11.80, CL 5.15, CW 4.10, OL 6.80, OW 5.10; ALE > PLE > PME > AME; palp 8.60 (3.05 + 1.50 + 1.95 + 2.10), leg I 10.50 (3.35 + 1.85 + 2.15 + 2.10 + 1.05), leg II 10.45 (3.40 + 1.75 + 2.00 + 2.20 + 1.10), leg III 10.95 (3.20 + 1.85 + 1.80 + 2.60 + 1.50), leg IV 15.70 (4.60 + 2.00 + 2.80 + 3.90 + 2.40).

***Female genitalia*.** A pair of depressions on the ventro-lateral part of the genital atrium (Fig. 21B, D). Paired receptacular clusters along the anterior margin of bursa copulatrix, divided into two parts, the inners longer than the laterals, both with several granules, without genital stalks (Fig. 21A, B).

**Male.** Unknown.

####### Etymology.

The species epithet, a noun in apposition, refers to the type locality.

####### Distribution.

The species is endemic to the Japanese island Tokashikijima (Fig. 1C).

###### Heptathela unten sp. nov.

Taxon classificationAnimaliaAraneaeLiphistiidae

5B74DB6F-F1ED-56B4-B4B2-02B6EFFA3D5D

http://zoobank.org/65FF6B26-074B-4DC8-87F8-021268D23D87

Figs 22 , 23

####### Type material.

***Holotype***: JAPAN · ♂; Okinawa-ken, Nakijin-son, Unten Port; 26.68N, 128.00E; alt. 25 m; 27 December 2012; D. Li, F.X. Liu and X. Xu leg.; XUX-2012-522.

***Paratypes***: JAPAN · 1 ♂, 2 ♀♀; same data as for holotype; XUX-2012-523, 527, 528A · 3 ♀♀; same data as for holotype; 10 May 2014; D. Li and B. Wu leg.; XUX-2014-083, 083A, 083B.

####### Diagnosis.

**Figure 22.**
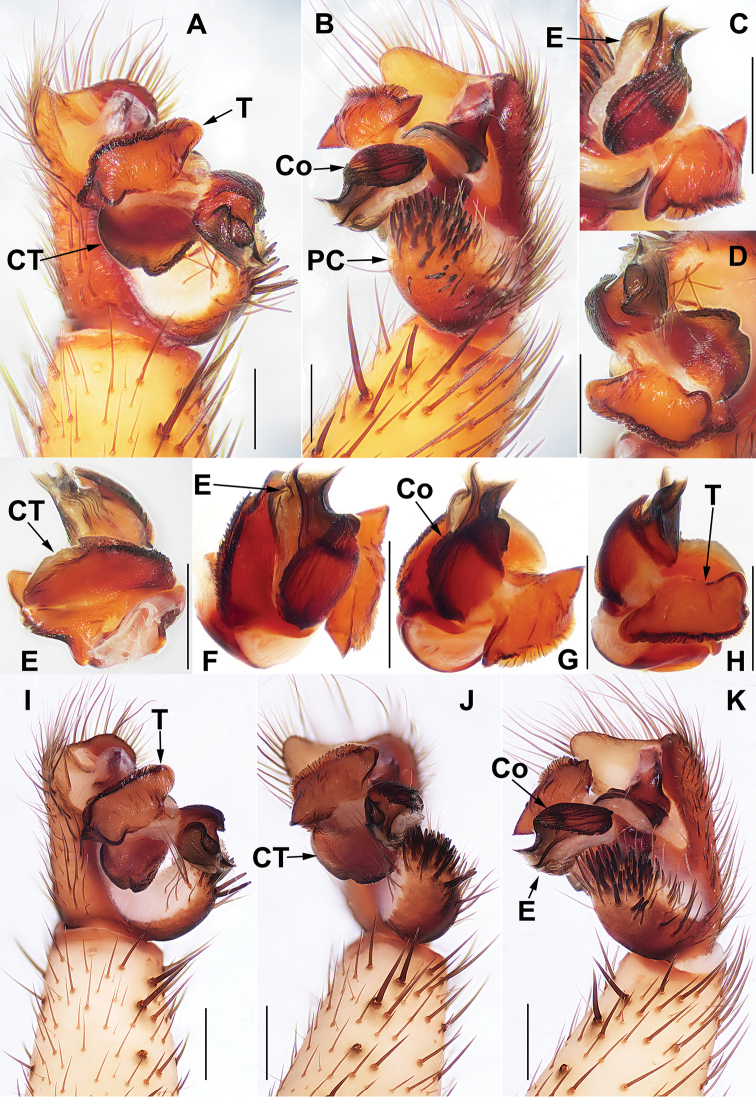
Male genital anatomy of *Heptathela unten* sp. nov. and *H. crypta* sp. nov. **A–D***Heptathela unten* sp. nov., 2522 (holotype, short for XUX-2012-522) **E–K***H. crypta* sp. nov. **E–H** 2328 (holotype) **I–K** 2460 **A, I** prolateral view **B, K** retrolateral view **J** ventral view **C–H** distal view; 2522: Unten Port, Okinawajima; 2328: Taira, Haneiji–Dam, Okinawajima; 2460: Yofuke, Okinawajima. Scale bar: 0.5 mm.

**Figure 23.**
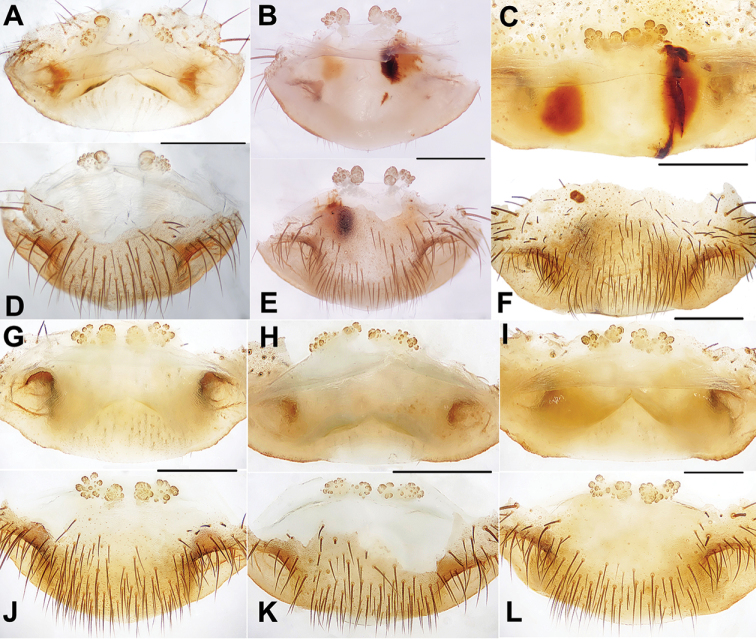
Female genital anatomy of *Heptathela unten* sp. nov. and *H. crypta* sp. nov. **A, D** 2527 **B, E** 4083A **C, F** 4027B **G, J** 2324 **H, K** 2327 **I, L** 2457 **A–C, G–I** dorsal view **D–F, J–L** ventral view; 2527, 4083A: Unten Port, Okinawajima; 4027B: Mt. Nago-dake, Okinawajima; 2324, 2327: Taira, Haneiji-Dam, Okinawajima; 2457: Yofuke, Okinawajima. Scale bar: 0.5 mm.

Males of *H. unten* sp. nov. can be distinguished from those of *H. yanbaruensis* by the blunt tegular marginal apophysis (Fig. 22A, C, D); from those of *H. helios* by the conductor with serrated margin and small tegular marginal apophysis (Fig. 22A, C). Females of *H. unten* sp. nov. cannot be distinguished morphologically form those of *H. crypta* sp. nov. (Fig. 23A–L). However, *H. unten* sp. nov. can be diagnosed from all other Okinawa group *Heptathela* species by the following unique nucleotide substitutions in the standard DNA barcode alignment: G (482), C (635).

####### Description.

**Male** (Holotype). Carapace yellow brown; opisthosoma brown, with dark brown tergites close to each other; cheliceral groove with ten denticles; seven spinnerets. Measurements: BL 10.00, CL 5.05, CW 4.60, OL 4.60, OW 2.90; ALE > PLE > PME > AME; leg I 15.71 (4.48 + 1.93 + 3.30 + 4.00 + 2.00), leg II 16.59 (4.45 + 1.90 + 3.37 + 4.62 + 2.25), leg III 18.29 (4.50 + 1.93 + 3.58 + 5.53 + 2.75), leg IV 23.40 (5.82 + 2.28 + 4.52 + 7.30 + 3.48).

***Palp*.** Prolateral side of paracymbium unpigmented and unsclerotised, numerous setae and spines at the tip of paracymbium (Fig. 22A, B). Contrategulum margin incurved nearly in the middle and the contrategulum divided into proximally serrated and distally smooth margins (Fig. 22A, D). Tegulum with wide dorsal extension of terminal apophysis (Fig. 22C, D), blunt terminal and small marginal apophysis (Fig. 22A, C, D). Conductor sclerotised and ovate, prolateral conductor with one or two shallow folds, and with a serrated margin (Fig. 22B, C). Embolus sclerotised, with a wide opening, the distal margin slightly sclerotised, and with a saddle-shaped margin in the retrolateral view (Fig. 22B, C).

**Females** (*N* = 5). Carapace and opisthosoma colour as in male, dark brown tergites separated from each other; cheliceral groove with 12 or 13 pronounced denticles; seven or eight spinnerets. Measurements: BL 7.81–12.00, CL 3.60–4.55, CW 3.30–4.40, OL 4.10–7.60, OW 3.00–6.20; ALE > PLE > PME > AME; palp 6.64 (2.19 + 1.22 + 1.45 + 1.78), leg I 7.48 (2.47 + 1.35 + 1.22 + 1.52 + 0.92), leg II 7.40 (2.21 + 1.30 + 1.21 + 1.65 + 1.03), leg III 7.79 (2.11 + 1.38 + 1.20 + 1.90 + 1.20), leg IV 11.55 (3.20 + 1.55 + 2.10 + 3.05 + 1.65).

***Female genitalia*.** A pair of depressions on the ventro-lateral part of genital atrium indistinct (Fig. 23D, E). Paired receptacular clusters along the anterior margin of bursa copulatrix, divided into two parts, the inners similar or smaller than the laterals, paired receptacular clusters tuberculate, inners with or without genital stalks (Fig. 23A, B, D, E).

####### Etymology.

The species epithet, a noun in apposition, refers to the type locality, Unten Port.

####### Distribution.

The species is endemic to the Japanese island Okinawajima (Fig. 1C).

###### Heptathela crypta sp. nov.

Taxon classificationAnimaliaAraneaeLiphistiidae

38088D00-4F7D-55FD-8F63-7E6FF5900E8B

http://zoobank.org/62DDDCB8-8B43-4EFC-B42C-9BAA1E114A23

Figs 22 , 23

####### Type material.

***Holotype***: JAPAN · ♂; Okinawa-ken, Nago-shi, Haneiji-Dam, Taira; 26.59N, 128.03E; alt. 100 m; 18 December 2012; D. Li, F.X. Liu and X. Xu leg.; XUX-2012-328.

***Paratypes***: JAPAN · 1 ♂, 3 ♀♀; same data as for holotype; XUX-2012-324, 326, 327, 333A · 2 ♂♂, 4 ♀♀; Okinawa Prefecture, Nago-shi, County Road 18 south, Nago/Yofuke; 26.57N, 128.01E; alt. 150 m; 24 December 2012; D. Li, F.X. Liu and X. Xu leg.; XUX-2012-457 to 462 · 3 ♀♀; Okinawa Prefecture, Nago-shi, Mt. Nago-dake; 26.58N, 128.01E; alt. 220 m; 06 May 2014; D. Li and B. Wu leg.; XUX-2014-027 to 027B.

####### Diagnosis.

Males and females of *H. crypta* sp. nov. cannot be distinguished morphologically from *H. unten* sp. nov. (Figs 22A–K, 23A–L), but can be diagnosed from *H. unten* sp. nov. by the following unique nucleotide substitutions in the standard DNA barcode alignment: C (26), A (32), C (50), T (60), T (110), G (153), T (194), C (197), T (269), C (281), T (284), C (338), A (341), T (357), C (416), T (428), C (458), A (482), T (488), G (551), T (581), T (635), G (638), G (641), C (644), C (656), as well as from all other Okinawa group *Heptathela* species by the following unique nucleotide substitutions in the standard DNA barcode alignment: C (26), T (110), G (551), C (656).

####### Description.

**Male** (Holotype). Carapace and opisthosoma description see *H. unten* sp. nov.; cheliceral groove with nine denticles of variable size; seven spinnerets. Measurements: BL 7.88, CL 4.01, CW 3.51, OL 4.23, OW 3.18; ALE > PLE > PME > AME; leg I 10.15 (3.60 + 1.50 + 2.38 + 1.00 + 1.67), leg II 13.08 (3.48 + 1.58 + 2.49 + 3.53 + 2.00), leg III 14.27 (3.38 + 1.55 + 2.65 + 4.30 + 2.39), leg IV 18.19 (4.45 + 1.63 + 3.50 + 5.50 + 3.11).

***Palp*.** Prolateral side of paracymbium unpigmented and unsclerotised, numerous setae and spines at the tip of paracymbium (Fig. 22I–K). Contrategulum margin incurved nearly in the middle, and the contrategulum divided into proximally serrated and distally smooth (Fig. 22E, F, I, J). Tegulum with wide dorsal extension of terminal apophysis (Fig. 22G, H, J), blunt terminal and small marginal apophysis (Fig. 22H, I). Conductor sclerotised and ovate, prolateral conductor with one or two shallow folds, and with a serrated margin (Fig. 22F, G, K). Embolus sclerotised, with a wide opening, the distal margin slightly sclerotised, and with a saddle-shaped margin in the retrolateral view (Fig. 22F, G, K).

**Females** (*N* = 10). Carapace and opisthosoma description see *H. unten* sp. nov.; chelicerae with promargin of cheliceral groove with 13–14 pronounced denticles of variable size; seven spinnerets. Measurements: BL 8.35–16.50, CL 4.07–5.10, CW 3.30–4.80, OL 4.70–6.80, OW 3.00–5.20; ALE > PLE > PME > AME; palp 7.70 (2.87 + 1.13 + 1.68 + 2.02), leg I 9.57 (3.07 + 1.70 + 1.70 + 1.98 + 1.12), leg II 9.64 (2.95 + 1.68 + 1.61 + 2.08 + 1.32), leg III 9.60 (2.68 + 1.69 + 1.50 + 2.30 + 1.43), leg IV 14.21 (4.00 + 1.92 + 2.55 + 3.83 + 1.91).

***Female genitalia*.** A pair of depressions on the ventro-lateral part of genital atrium indistinct (Fig. 23F, J–L). Paired receptacular clusters along the anterior margin of bursa copulatrix, divided into two parts, the inners similar or smaller than the laterals, paired receptacular clusters tuberculate, without genital stalks (Fig. 23C, G–L).

####### Etymology.

The species epithet, a noun in apposition, refers to the cryptic nature of this species discovery.

####### Distribution.

The species is endemic to the Japanese island Okinawajima (Fig. 1C).

##### Species not assigned to a group

###### Heptathela helios

Taxon classificationAnimaliaAraneaeLiphistiidae

Tanikawa & Miyashita, 2014

D5DE01B4-97B9-536E-ADD0-1443A190F9B8

Fig. 24

Heptathela helios Tanikawa & Miyashita, 2014: 68 (holotype: male (NSMT-Ar 12851), from Kunigami-son, Okinawajima, Japan, collected by A. Tanikawa on 26 May 2010, matured on 9 September 2012, deposited in NMNS, examined).

####### Diagnosis.

**Figure 24.**
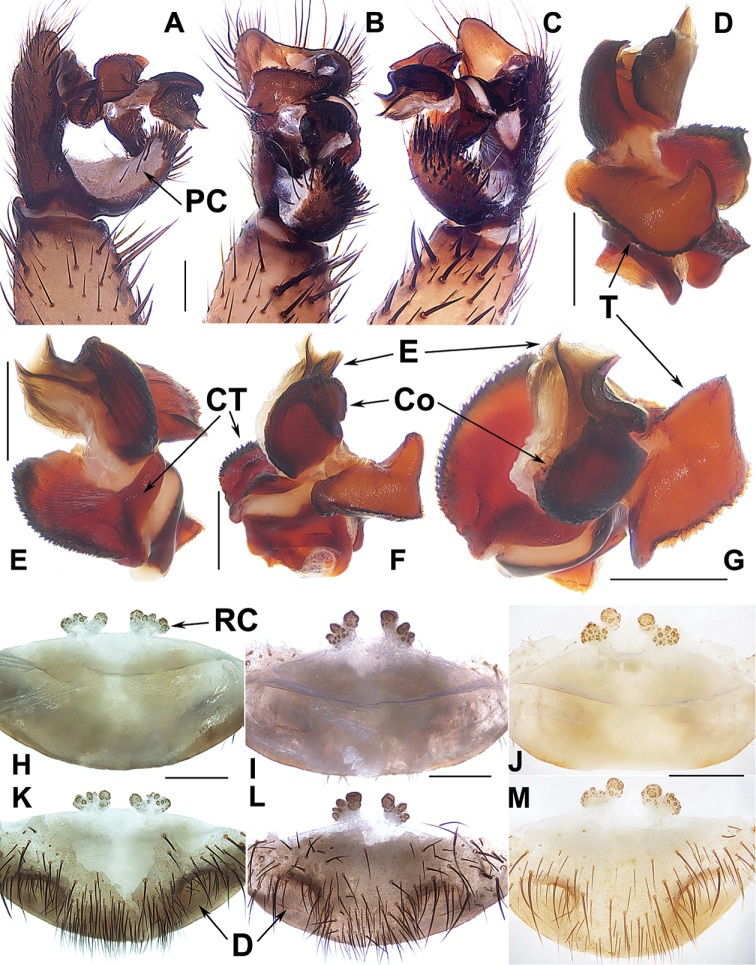
Male and female genital anatomy of *Heptathela helio* Tanikawa & Miyashita, 2014 **A–G** 2440A (short for XUX-2012-440A) **H, K** 2432 **I, L** 2434 **J, M** 2437 **A** palp prolateral view **B** palp ventral view **C** palp retrolateral view **D–G** palp distal view **H–J** vulva dorsal view **K–M** vulva ventral view. Scale bar: 0.5 mm.

Males of *H. helios* can be distinguished from those of all other Okinawa group *Heptathela* species by the serrated contrategulum margin and the hooked tegular marginal apophysis, the ovate, indistinctly rugose conductor with a poorly serrated margin (Fig. 24D–G). Females of *H. helios* can be distinguished from those of all other *Heptathela* species by the receptacular clusters with the inner ones being smaller than the laterals, and the laterals with numerous small granulate tubercula (Fig. 24H–M). *H. helios* can also be diagnosed from all other Okinawa group *Heptathela* species by the following unique nucleotide substitutions in the standard DNA barcode alignment: T (11), T (35), G (47), A (56), C (59), A (95), T (104), A (131), T (140), T (179), C (188), C (215), G (221), C (242), C (266), C (273), C (299), C (300), C (304), T (359), G (380), T (413), A (422), T (425), T (431), G (479), G (480), G (491), G (506), C (543), T (546), G (548), C (551), C (596), T (662).

####### Description.

**Male.** Carapace and opisthosoma brown, with dark brown tergites; cheliceral groove with 14 denticles; tergites closed to each other; seven spinnerets. Measurements: BL 9.88, CL 5.30, CW 4.81, OL 4.58, OW 2.92; ALE > PLE > PME > AME; leg I 16.56 (4.55 + 1.90 + 3.48 + 4.33 + 2.30), leg II 17.19 (4.50 + 2.00 + 3.34 + 4.68 + 2.67), leg III 18.35 (4.35 + 1.97 + 3.40 + 5.53 + 3.10), leg IV 23.37 (5.52 + 2.10 + 4.47 + 7.48 + 3.80).

***Palp*.** The bulb of the two male specimens relatively distorted. Prolateral side of paracymbium unpigmented and unsclerotised, numerous setae and spines at the tip of paracymbium (Fig. 24A–C). Contrategulum with a serrated margin (Fig. 24E, G). Tegulum with a dentate dorsal extension of terminal apophysis (Fig. 24F, G), blunt terminal and hook-like marginal apophysis (Fig. 24D, F, G). Conductor sclerotised, ovate, and wide, with indistinct rugae (Fig. 24E–G). Embolus sclerotised, with a wide opening, the distal margin slightly sclerotised, and with a saddle-shaped margin in the retrolateral view (Fig. 24D–G).

**Females** (*N* = 7). Carapace and opisthosoma colour as in male; cheliceral groove with 12–14 pronounced denticles; opisthosoma with 12 well-separated tergites; seven spinnerets. Measurements: BL 11.70–14.45, CL 5.29–6.70, CW 4.29–5.81, OL 6.54–8.18, OW 4.70–6.63; ALE > PLE > PME > AME; palp 12.29 (4.20 + 2.03 + 2.63 + 3.43), leg I 14.31 (4.51 + 2.40 + 2.65 + 3.08 + 1.67), leg II 14.04 (4.25 + 2.35 + 2.53+ 3.11 + 1.80), leg III 13.75 (3.92 + 2.40 + 2.48 + 2.67 + 2.28), leg IV 21.77 (5.90 + 2.88 + 3.75 + 6.11 + 3.13).

***Female genitalia*.** A pair of depressions on the ventro-lateral part of genital atrium (Fig. 24K–M). Paired receptacular clusters along the anterior margin of bursa copulatrix, divided into two parts, inner receptacular clusters smaller than laterals, laterals with several small tubercula, with short genital stalks (Fig. 24H–M).

####### Remarks.

We identified the specimens collected from Ginama Dam, Okinawa, as *H. helios* based on evidence from morphology and COI barcode genetic distance compared with the male holotype and paratype (NSMT-Ar 12851, NSMT-Ar 12855) of *H. helios* in Tanikawa and Miyashita (2014). K2P and *p*-distances between Ginama Dam specimens and the holotype (NSMT-Ar 12851) were 3.2–3.4% and 3.1–3.3%, respectively, and those between Ginama Dam specimens and the paratype (NSMT-Ar 12855) were 1.8–2.1% and 1.7–2.1%, respectively.

####### Material examined.

JAPAN · 2 ♂♂, 8 ♀♀; Okinawa-ken, Kunigami-son, Ginama Dam; 26.84N, 128.26E; alt. 150 m; 24 December 2012; D. Li, F.X. Liu and X. Xu leg.; XUX-2012-432 to 440C.

####### Distribution.

The species is endemic to the Japanese island Okinawajima (Fig. 1C).

## Supplementary Material

XML Treatment for Heptathela higoensis

XML Treatment for Heptathela kikuyai

XML Treatment for Heptathela kimurai

XML Treatment for Heptathela yakushimaensis

XML Treatment for Heptathela amamiensis

XML Treatment for Heptathela kanenoi

XML Treatment for Heptathela kojima

XML Treatment for Heptathela sumiyo

XML Treatment for Heptathela uken

XML Treatment for Heptathela yanbaruensis

XML Treatment for Heptathela aha

XML Treatment for Heptathela gayozan

XML Treatment for Heptathela kubayama

XML Treatment for Heptathela mae

XML Treatment for Heptathela otoha

XML Treatment for Heptathela shuri

XML Treatment for Heptathela tokashiki

XML Treatment for Heptathela unten

XML Treatment for Heptathela crypta

XML Treatment for Heptathela helios

## Supplementary materials

Supplementary material 1 Table S1 Data type: species data https://binary.pensoft.net/file/354516This dataset is made available under the Open Database License (http://opendatacommons.org/licenses/odbl/1.0/). The Open Database License (ODbL) is a license agreement intended to allow users to freely share, modify, and use this Dataset while maintaining this same freedom for others, provided that the original source and author(s) are credited.Xin Xu, Hirotsugu Ono, Matjaž Kuntner, Fengxiang Liu, Daiqin Li
